# HMGCLL1 is a predictive biomarker for deep molecular response to imatinib therapy in chronic myeloid leukemia

**DOI:** 10.1038/s41375-018-0321-8

**Published:** 2018-12-16

**Authors:** Jong-Ho Park, Young Min Woo, Emilia Moonkyung Youm, Nada Hamad, Hong-Hee Won, Kazuhito Naka, Eun-Ju Park, June-Hee Park, Hee-Jin Kim, Sun-Hee Kim, Hyeoung-Joon Kim, Jae Sook Ahn, Sang Kyun Sohn, Joon Ho Moon, Chul Won Jung, Silvia Park, Jeffrey H. Lipton, Shinya Kimura, Jong-Won Kim, Dennis (Dong Hwan) Kim

**Affiliations:** 1 Department of Health Sciences and Technology, Samsung Advanced Institute for Health Sciences and Technology, Sungkyunkwan University, Seoul, Korea; 2 Department of Haematology, St Vincent’s Hospital, University of New South Wales, Sydney, Australia; 3 Samsung Advanced Institute for Health Sciences and Technology, Sungkyunkwan University, Samsung Medical Center, Seoul, Korea; 4 Department of Stem Cell Biology, Research Institute for Radiation Biology and Medicine, Hiroshima University, Hiroshima, Japan; 5 Research Institute for Future Medicine, Samsung Medical Center, Seoul, Korea; 6 Department of Laboratory Medicine and Genetics, Samsung Medical Center, Sungkyunkwan University School of Medicine, Seoul, Korea; 7 Department of Hematology-Oncology, Chonnam National University Hwasun Hospital, Hwasun, Korea; 8 Department of Hematology/Oncology, Kyungpook National University Hospital, Daegu, Korea; 9 Department of Hematology/Oncology, Samsung Medical Center, Sungkyunkwan University School of Medicine, Seoul, Korea; 10 Department of Medical Oncology & Hematology, Princess Margaret Cancer Centre, University Health Network, University of Toronto, Toronto, Canada; 11 Division of Hematology, Respiratory Medicine and Oncology, Department of Internal Medicine, Faculty of Medicine, Saga University, 5-1-1 Nabeshima, Saga, 849-8501 Japan

**Keywords:** Chronic myeloid leukaemia, Pharmacogenomics, Cancer genetics

## Abstract

Achieving a deep molecular response (DMR) to tyrosine kinase inhibitor (TKI) therapy for chronic myeloid leukemia (CML) remains challenging and at present, there is no biomarker to predict DMR in this setting. Herein, we report that an *HMGCLL1* genetic variant located in 6p12.1 can be used as a predictive genetic biomarker for intrinsic sensitivity to imatinib (IM) therapy. We measured DMR rate according to *HMGCLL1* variant in a discovery set of CML patients (*n* = 201) and successfully replicated it in a validation set (*n* = 270). We also investigated the functional relevance of *HMGCLL1* blockade with respect to response to TKI therapy and showed that small interfering RNA mediated blockade of *HMGCLL1* isoform 3 results in significant decrease in viability of *BCR-ABL1*-positive cells including K562, CML-T1 or BaF3 cell lines with or without *ABL1* kinase domain mutations such as T315I mutation. Decreased cell viability was also demonstrated in murine CML stem cells and human hematopoietic progenitor cells. RNA sequencing showed that blockade of *HMGCLL1* was associated with G0/G1 arrest and the cell cycle. In summary, the *HMGCLL1* gene polymorphism is a novel genetic biomarker for intrinsic sensitivity to IM therapy in CML patients that predicts DMR in this setting.

## Introduction

Introduction of tyrosine kinase inhibitors (TKI) has revolutionized the clinical management of patients with chronic myeloid leukemia (CML) [1] and has significantly improved the life expectancy of this group of patients to up to 98% of that in the general population [2]. In patients who achieve a sustained deep molecular response (DMR; defined as a molecular response with 4 or 4.5-log reduction) with TKI therapy, discontinuation of TKI therapy has emerged as a potential treatment option [3, 4]. However, early identification of patients with higher chances of achieving DMR remains challenging and no predictive biomarker of DMR following TKI therapy has so far been described in CML patients.

Intrinsic drug sensitivity to TKI therapy is widely variable among CML patients. Although ~ 70% of CML patients can achieve optimal responses such as major molecular response (MMR) following TKI therapy, not all of them can achieve DMR. Also the speed to achieve DMR is very variable. Some patients can rapidly achieve DMR typically within a year or two following initiation of TKI therapy, whereas others cannot achieve it even with longer durations of treatment. Given the observed wide spectrum of intrinsic sensitivity to TKI therapy among patients with CML, identification of a genetic biomarker that can predict DMR following TKI therapy could be of clinical value [5].

In the present study, we performed a genome-wide association study (GWAS) to identify genetic markers associated with DMR. We propose that *HMGCLL1* gene polymorphism is a novel predicative biomarker for DMR following imatinib (IM) therapy. We also demonstrate that *HMGCLL1* polymorphism is functionally relevant for CML cell growth and viability, and that *HMGCLL1* blockade is cytotoxic to CML cells.

## Material and methods

### Discovery and validation data sets

We performed a GWAS on peripheral blood samples from 202 CML patients with East Asian ethnicity as a discovery set. The discovery set had been utilized in a previous study to identify a germline polymorphism marker associated with increased susceptibility to CML. A separate set of samples from 272 CML patients of European descent recruited in Canada was used as validation set. All patients in the discovery and validation sets were treated with IM frontline therapy [6–9, 40]. The study was approved by the Institutional Review Boards.

### Genotyping and quality control in the discovery and validation sets

In the discovery set, 906,530 SNPs were genotyped using Genome-Wide Human SNP Array 6.0 (Affymetrix, Santa Clara, CA, USA). SNPs showing erroneous genotype clustering patterns were filtered out. One sample with a missing genotype rate of > 5% was excluded from the analysis. In addition, 39,033 SNPs were excluded owing to low genotyping (with > 5% missing genotypes per marker) and 198,553 SNPs, owing to minor allele frequency of < 1%. A total of 637,886 autosomal SNPs in the discovery set (*n* = 201) were included in the final analysis.

We evaluated treatment response parameters following IM therapy including complete cytogenetic response (CCyR), and DMR. Definition of response following IM therapy is described in the Supplementary Information. Candidate loci associated with DMR were selected with the following criteria: minimum *p* values of < 5.0 × 10^–5^, and > five SNPs with *p* < 10^−4^ within 1 Mb. For validation, a total of 25 SNPs with *p* < 10^−4^ in candidate regions (i.e., 6p12.1 and 16q23.2) were genotyped in the validation set using the MassARRAY iPLEX system (Agena Bioscience, San Diego, CA, USA). Two samples with high missing genotype rates ( > 50%) were excluded from the final analysis.

### Bioinformatics analysis

Linkage disequilibrium structure was assessed using Haploview version 4.2.7 [10]. IMPUTE2 program version 2.3.0 was used to impute SNPs not covered by Affymetrix SNP array 6.0 [11, 12]. We also used a pre-phasing method, SHAPEIT2 (version v2.r727), for small loss in accuracy with fast imputation [13, 14]. The reference panel used for imputation comprised of 90 known JPT + CHB haplotypes from the International HapMap Project data (Phase II Public Release #22 NCBI Build 36) [15]. Genome coordinates were converted from hg18 to hg19 using LiftOver tool from UCSC browser [16]. LocusZoom software was used to depict candidate regions in detail [17].

### Fine mapping and expression quantitative trait loci (eQTL) analysis

Fine mapping and eQTL analysis were performed as described in the Supplementary Information.

### Functional analysis of *HMGCLL1* based on in vitro methods

We performed functional analysis of *HMGCLL1* in order to investigate the effects of *HMGCLL1* isoform type 3 blockade on cell lines expressing *BCR-ABL1*, as well as on hematopoietic progenitor cells (HPCs) isolated from CML patients’ samples. Phospho-CrkL (pCrkL) assay and cell cycle analysis were also conducted. We performed more than three independent experiments with at least triplicates to ensure accuracy. Detailed descriptions of *in vitro* experiments are described in the Supplementary Information.

### Statistical analysis

Cumulative incidence of responses to IM therapy including CCyR, MMR, and DMR were calculated considering competing risks (i.e., switch to other TKI or death or progression). Gray’s test was used for comparison according to TCGAATAC haplotype. The Fine-Gray model was adopted for multivariate analysis. Student’s *t* test was used for independent samples, and the Wilcoxon rank sum or Kruskal–Wallis rank sum test was used to calculate difference in cell viability or for eQTL analysis. All statistical analyses were performed using PLINK Version 1.07 [41], R (R Foundation for Statistical Computing, Austria), and EZR software (https://www.jichi.ac.jp/saitama-sct/SaitamaHP.files/statmedEN.html) [18].

## Results

### GWAS identified a locus of 6p12.1 as a predictive marker for DMR following IM therapy

**Fig. 1:**
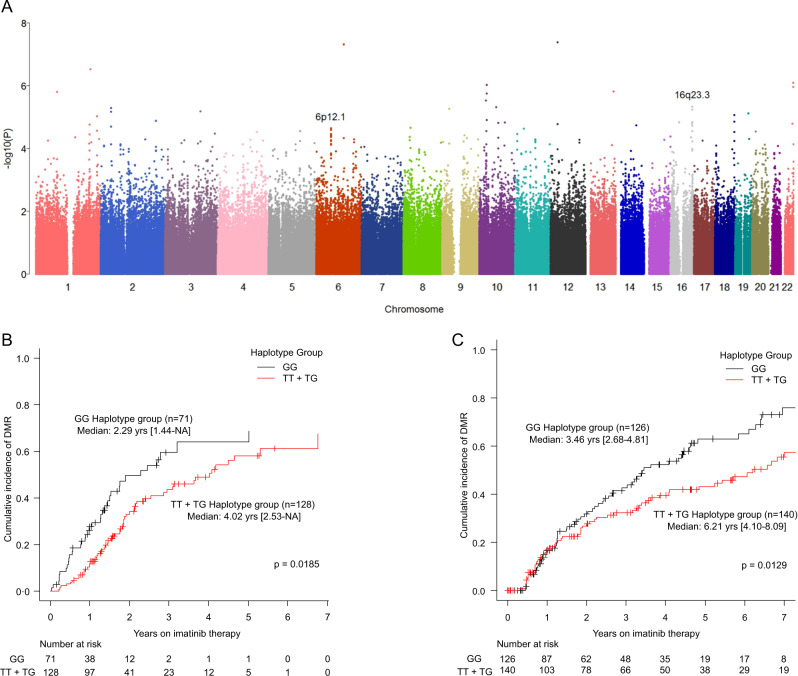
Results of genome-wide association analysis. **a** Manhattan plot shows the genome-wide *p* value identified in the discovery set of 201 chronic myeloid leukemia (CML) patients following imatinib (IM) therapy. Two loci (i.e., 6p12.1 and 16q23.2) were selected as candidate loci, each including more than five SNPs with *p* < 10^−4^ within 1 Mb and minimum *p* values of less than 5.0 × 10^−5^. **b** and **c** The plots show cumulative incidence of deep molecular response (DMR; defined as a molecular response with 4 or 4.5-log reduction) in the discovery and validation sets, respectively. The red line indicates the group with TCGAATAC. TT indicates TCGAATAC/TCGAATAC homozygote haplotype. TG represents TCGAATAC/GTCTGCGT heterozygote haplotype. GG indicates GTCTGCGT/GTCTGCGT homozygote haplotype. Two cases in the discovery set and three cases in the validation set did not have haplotype information due to missing data of genotype or different haplotype constructed. One case in the validation set had missing data on days of achieving DMR

In the discovery set of CML patients (*n* = 201), two loci (i.e., 6p12.1 and 16q23.3) were identified as potential candidates associated with DMR based on our selection criteria (Fig. 1a; Supplementary Fig. 1; Supplementary Table 1). Minimum *p* values of 2.25 × 10^−5^ for 6p12.1 and 4.64 × 10^−6^ for 16q23.3 were observed. Candidate genes included *HMGCLL1, GFRAL*, and *BMP5* near the 6p12.1 locus and *CDH13* and *HSBP1* near the 16q23.3 locus.

In the validation set (*n* = 270), we attempted to replicate these two candidate loci (6p12.1 and 16q23.3) that were identified in the discovery set. Demographics and disease characteristics of patients in the discovery/validation sets are summarized in Table 1. The locus 6p12.1 was successfully validated, including eight SNPs (rs10948926, rs10948927, rs9370435, rs4546489, rs4275061, rs9475323, rs9475327, and rs9296791; Table 2), all of which were located in *HMGCLL1* intron 6 (between exon 6 and exon 7). These SNPs showed high linkage disequilibrium in the same block (*r*^2^ ≥ 0.99; Supplementary Fig. 2). As a representative SNP, the frequency of G allele in rs9370435 in the responder group from the discovery set was lower than that in the non-responder group (0.37 vs 0.45). A similar trend was seen in the validation set (0.29 vs 0.35). We also evaluated the association of eight SNPs, which were previously validated as markers for DMR following IM therapy with MMR and CCyR parameters (Supplementary Tables 2–4).

**Table 1:** Demographic and disease characteristics of patients treated with imatinib for chronic myeloid leukemia in chronic phase

Patient’s characteristics	No. of Pts (%)	Discovery set			Validation set			*p* value^a^
		Overall	TCGAATAC	Non-TCGAATAC	Overall	TCGAATAC	Non-TCGAATAC	
No of pts	471	201^b^	128	71	270^b^	141	126	0.015
Age (median, year)	52 (15–82)	50 (15–81)	51 (15–78)	48 (18–81)	54.5 (21–82)	57 (21–82)	52 (21–81)	
Gender (female:male)	195/276 (41:59)	81/120 (40:60)	49/79 (38:62))	31/40 (44:56)	114/156 (42:58)	56/85 (40:60)	57/69 (45:55)	0.675
Disease stage (CP:AP:BC)	423/42/6 (90:9:1)	182/17/2 (90:9:1)	114/13/1 (89:10:1)	67/3/1 (94:4:1)	241/25/4 (89:9:2)	129/10/2 (92:7:1)	110/14/2 (87:11:2)	0.853
Sokal risk group (Low:Intermediate:High)	99/126/89 (31:40:28)	52/81/46 (29:45:26)	29/60/23 (26:54:21)	23/21/21 (35:32:32)	47/45/43 (35:33:32)	25/24/22 (35:34:31)	22/21/20 (35:33:32)	0.102
Additional chromosomal abnormalities*	53 (11)	21 (10)	9 (7)^c^	11 (16)^d^	32 (12)	16 (11)^e^	16 (13)^f^	0.633

*CP*, chronic phase, *AP* accelerated phase, *BC* blastic crisis

^a^ Between discovery set and validation set

^b^ Two cases in the discovery set and three cases in the validation set are missing for haplotype due to some missing data of genotype or different haplotype constructed

^c^ TCGAATAC haplotype group in the discovery set: -12 (*n* = 1); del (22) (*n* = 1); t(9;22;17) (*n* = 1); double Ph chromosome (*n* = 1); inv (9), der (22) (*n* = 1); t(2;9;22) (*n* = 1); t(4;17) (*n* = 1); t(5;9;22) (*n* = 1); t(9;22;11) (*n* = 1)

^d^ Non-TCGAATAC haplotype group in the discovery set: -21. + 22 (*n* = 1); -Y (*n* = 1); t(13;22) (*n* = 1); inv(3) (*n* = 1); dup(1) (*n* = 1); t(13;14) (*n* = 1); + 8, + der(22) (*n* = 1); inv(9) (*n* = 1); t(4;22), t(17;20) (*n* = 1); t(9;22;13) (*n* = 1); triploid to hexaploid (*n* = 1)

^e^ TCGAATAC haplotype group in the validation set: -Y (*n* = 4); inv (11) (*n* = 1); double Ph chromosome (*n* = 4); t(2;9;22) (*n* = 1); t(4;6), + X, + 6, + 8, + 18, + 19, + der(22) (*n* = 1); t(9;17), t(5(22); t(17;22), t(15;22) (*n* = 1); t(9;22;17) (*n* = 1); t(9;22;22) (*n* = 1); t(8;17) (*n* = 1); t(8;16) (*n* = 1)

^f^ Non-TCGAATAC haplotype group in the validation set: -X(*n* = 1); -Y(*n* = 2); + 8(*n* = 1); del 18q (*n* = 1); double Ph chromosome (*n* = 4); t(17;20), der (17), der (20) (*n* = 1); t(1;22;18), inv (5) (*n* = 1); t(12;16) (*n* = 1); t(3;19) (*n* = 1); t(7;8), + 8, + der(22) (*n* = 1); t(9;22;22) (*n* = 2)

**Table 2:** List of eight SNPs significantly associated with deep molecular response achievement following imatinib therapy in chronic myeloid leukemia patients

Chromosome	SNP	Position^a^	Minor allele	Major allele	Set	MAF in Response	MAF in Non-response	*p* value	HR (CI, 95%)
6	rs10948926	55366347	T	G	Discovery	0.38	0.45	7.47E-05	0.54 (0.4–0.73)
					Validation	0.29	0.35	0.048	0.761 (0.581–0.997)
6	rs10948927	55366392	C	T	Discovery	0.38	0.45	7.47E-05	0.54 (0.4–0.73)
					Validation	0.29	0.35	0.048	0.761 (0.581–0.997)
6	rs9370435	55369916	G	C	Discovery	0.37	0.45	3.48E-05	0.52 (0.38–0.71)
					Validation	0.29	0.35	0.037	0.749 (0.57–0.982)
6	rs4546489	55370236	A	T	Discovery	0.37	0.45	4.21E-05	0.53 (0.39–0.71)
					Validation	0.29	0.35	0.049	0.764 (0.584–0.999)
6	rs4275061	55371577	A	G	Discovery	0.38	0.45	7.47E-05	0.54 (0.4–0.73)
					Validation	0.29	0.35	0.041	0.754 (0.575–0.988)
6	rs9475323	55371939	T	C	Discovery	0.38	0.45	7.47E-05	0.54 (0.4–0.73)
					Validation	0.29	0.35	0.048	0.761 (0.581–0.997)
6	rs9475327	55372270	A	G	Discovery	0.38	0.45	7.61E-05	0.54 (0.4–0.73)
					Validation	0.29	0.35	0.048	0.761 (0.581–0.997)
6	rs9296791	55376167	C	T	Discovery	0.38	0.45	7.47E-05	0.54 (0.4–0.73)
					Validation	0.29	0.35	0.041	0.754 (0.575–0.988)

*HR* hazard ratio, *CI* confidence interval, *MAF* minor allele frequency

^a^ Physical position based on human reference genome build hg19 (GRCh37)

In the discovery and validation sets, two representative haplotypes were constructed based on these eight SNPs (TCGAATAC and GTCTGCGT). These haplotypes covered 99% of samples in both the discovery and validation sets. The TCGAATAC haplotype was associated with lower chances of achieving DMR both in the discovery set (hazard ratio [HR]: 0.52; 95% confidence interval (0.40–0.73)) and the validation set (HR: 0.75 (0.57–0.98)) (Table 2). When the cumulative incidence of DMR was analyzed, the group with the TCGAATAC haplotype showed a lower DMR rate than the group without this haplotype both in the discovery (Fig. 1b; Supplementary Fig. 3a) and validation sets (Fig. 1c; Supplementary Fig. 3b). When analyzed for MMR, as shown in Supplementary Fig. 4, the group with TCGAATAC haplotype showed a lower MMR rate compared with those without this haplotype both in the discovery and validation sets.

In multivariate analysis, the group with TCGAATAC haplotype had a lower probability of achieving DMR following IM therapy as expected. TCGAATAC haplotype remained an independent risk factor for DMR both in the discovery (*p* *=* 0.044, HR: 0.65 (0.43–0.99)) and validation sets (*p* *=* 0.019, HR: 0.66 (0.47–0.93)) after adjusting for variables such as age, disease stage, gender, and additional cytogenetic aberrations.

In order to exclude the possibility that these SNPs associate with CML leukemogenesis, we examined their frequency between CML patients and healthy controls. Minor allele type (i.e., TCGAATAC haplotype or G allele in rs9370435) frequency in CML patients showed similar frequency to control subjects (0.41 vs 0.39; Supplementary Table 5), implying that the SNPs are not associated with susceptibility to CML.

### Fine mapping study reveals that *HMGCLL1* polymorphism haplotypes correlates with DMR

In order to find potential causal variants associated with a risk haplotype, we performed targeted re-sequencing of the *HMGCLL1* gene [19]. We selected 30 cases of CML (15 from the discovery and 15 from the validation group). Of these patients, 16 achieved DMR, whereas 14 did not (Supplementary Table 6). To maximize the detection probability of a DMR-associated functional variant, we excluded the cases carrying heterogeneous haplotypes and only included cases that carried a homogeneous haplotype of TCGAATAC (adverse haplotype) or GTCTGCGT (referent haplotype) [20]. A total of 673 variants (626 SNPs and 47 indels) were detected from the discovery set and 980 variants (869 SNPs and 111 indels) from the validation set. Although a coding variant was not detected in targeted re-sequencing, a few SNPs were found to be located in regulatory elements of *HMGCLL1* gene, such as DNase I hypersensitive site (DHS) or in a sensitive region as previously described by Khurana et al. [21, 22]. Specifically, in intron 6 where the TCGAATAC haplotype is located, three SNPs (rs7769320, rs7751266, and rs4521577) overlapped with the sensitive region and DHS in both sets. This finding implies that the intron 6 region is an important regulatory site that may affect gene expression and transcript splicing of *HMGCLL1* through genetic variations, such as TCGAATAC haplotype [23].

### *HMGCLL1* produces several subtypes of *HMGCLL1* isoforms

*HMGCLL1* is known to be transcribed into various alternative isoforms. To understand the functional role of the haplotype, we performed eQTL analysis to investigate the expression of *HMGCLL1* isoforms [24]. The eQTL has been broadly utilized to investigate the biological effects of causal variants located in non-coding regions [25]. Detailed isoform types of the *HMGCLL1* gene were extracted from public databases (NCBI and Ensembl; Supplementary Table 7). Isoforms were named from IS1 to IS6 according to the exon count of the isoforms. Six variants with total gene products spanning the whole *HMGCLL1* gene region, IS1 + IS2, IS3 + IS6, IS4, IS5, and IS6, were found at mRNA level in normal samples (Supplementary Fig. 5; Supplementary Table 8). Of note, eQTL signals were significantly associated with TCGAATAC haplotype in the total gene and in isoform IS3 (Total Gene: *p* *=* 0.0098, IS3 + IS6: *p* *=* 0.024; Supplementary Fig. 5b). The genotypic model also confirmed a significant association between total gene expression and TCGAATAC haplotype (Total Gene: *p* *=* 0.015; Supplementary Fig. 5c). Subjects with TCGAATAC haplotype showed higher gene expression levels of *HMGCLL1* compared with those with GTCTGCGT haplotype in IS3, suggesting that *HMGCLL1* gene polymorphism affects the expression level of a specific isoform [26]. Based on this result, we further investigated the effects of *HMGCLL1* blockade targeting IS3 on CML cell lines as well as HPCs.

### IS3 small interfering RNA reduces cell growth in IM-sensitive and IM-resistant BaF3 cell lines

**Fig. 2:**
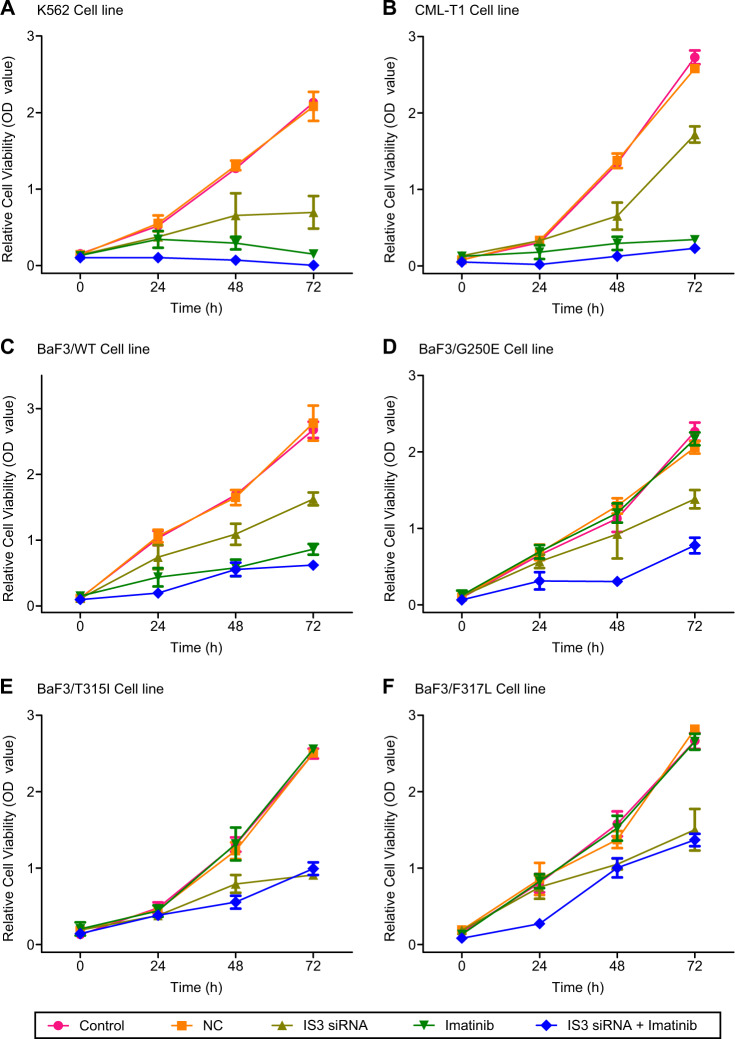
Results of cell viability assay using K562, CML-T1, and BaF3 cell lines with *ABL1* kinase domain mutations. **a** K562, **b** CML-T1, **c** BaF3/WT, **d** BaF3/G250E^*mut*^, **e** BaF3/T315I^*mut*^, and **f** BaF3/F317L^*mut*^. IS3-targeted small-interfering RNA (IS3 siRNA) reduced growth of K562, CML-T1, BaF3/WT, BaF3/G250E^*mut*^, BaF3/T315I^*mut*^, and BaF3/F317L^*mut*^ cells. Results of cell growth are presented as average ± standard deviation of optical density (OD) value (*Y* axis) as time progresses (*X* axis) from at least three independent wells. Cell viability was also determined for untreated control, negative control (NC) treated with non-silencing siRNA, IS3si-treated, IM-treated, and combination of IS3si with IM-treated cells. Statistical analysis was performed using Student’s *t* test with equal variance at 72 h

To determine the effects of IS3 blockade on CML growth, K562 cells were transfected with small interfering RNAs targeting IS3 (IS3si) or negative controls and incubated with IM. Cell viability was measured using WST-8 assay 72 h after transfection. Compared with controls, cell viability (%mean ± SD) was significantly reduced to 32.59 ± 10.01 in the group transfected with IS3si, 7.11 ± 2.35 in the group treated with IM, and 0.17 ± 0.38 in the group treated with both IM and IS3si (Fig. 2a). Bliss independence (BLISS) analysis [27] showed a synergistic effect for IM therapy and IS3si in reducing the viability of K562 cells compared with IM alone.

Next, we evaluated the effect of blockade of other *HMGCLL1* isoforms on K562 cell line. Cells were transfected with siRNAs targeting various *HMGCLL1* isoforms (Supplementary Fig. 6a; Supplementary Table 9). The results of eQTL signal (i.e., IS3) and siRNA-targeted blockade of *HMGCLL1* isoforms were concordant and showed that expression level of *HMGCLL1* gene is strongly affected by the siRNA specific for isoform 3 (Supplementary Fig. 6b). TCGAATAC haplotype was associated with high IS3 expression level, which was associated with reduced DMR rate with IM therapy in the GWAS. Also, IS3 blockade inhibited the growth of K562 cells in vitro. Another *BCR-ABL1-*positive *(BCR-ABL1*^+^) cell line, CML-T1, also showed a similar trend of decrease in cell viability after treatment with IS3si, IM, or both (Fig. 2b).

**Fig. 3:**
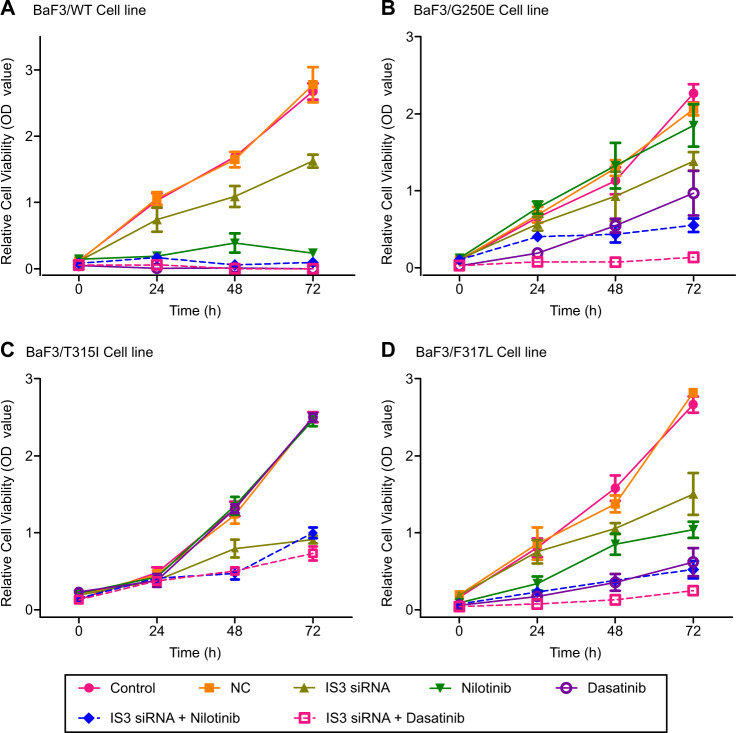
Results of cell viability assay after treatment with IS3si, dasatinib, and nilotinib in BaF3 cell lines with *ABL1* kinase domain mutations. **a** BaF3/WT, **b** BaF3/G250E^*mut*^, **c** BaF3/T315I^*mut*^, and **d** BaF3/F317L^*mut*^. IS3si reduced growth of BaF3/WT, BaF3/G250E^*mut*^, BaF3/T315I^*mut*^, and BaF3/F317L^*mut*^ cells. Results of cell growth are presented as average ± standard deviation of OD value (*Y* axis) as time progresses (*X* axis) from at least three independent wells. Cell viability was determined for untreated control, NC treated with non-silencing siRNA, IS3si-treated, dasatinib- or nilotinib-treated, and combination of IS3si with dasatinib- or nilotinib-treated cells. Statistical analysis was performed using Student’s *t* test with equal variance at 72 h

We next tested the effects of IS3 blockade on growth of BaF3 wild-type and IM-resistant *BCR-ABL1* mutant cells. Sequence homology of IS3 cDNA between human and murine genomes by ClustalW2 Multiple Sequence Alignment is known to be 88.35% [28]. The IS3si binding site sequence showed a perfect match between human and murine (Supplementary Fig. 6c), which enabled us to test murine-derived BaF3 cells using the protocols for human cell lines. As expected, all those cell lines (BaF3/G250E^*mut*^, BaF3/T315I^*mut*^, and BaF3/F317L^*mut*^) showed resistance to IM treatment (Fig. 2c–f). After treatment with IS3si alone, viabilities (%mean ± SD) of these three cell lines were reduced to 60.21 ± 5.72, 36.45 ± 1.98, and 56.40 ± 10.20, respectively. When IM was added to IS3si treatment, the repression of cell viability was observed regardless of the presence of mutations within *ABL1* kinase domain (Fig. 2d–f). In wild-type BaF3 cells, synergistic inhibition of cell viability was observed when second generation TKIs, nilotinib, or dasatinib, were combined with IS3si (Fig. 3a). Dasatinib reduced cell growth to 23.17 ± 6.82% in BaF3/F317L^*mut*^ and to 42.70 ± 12.77% in BaF3/G250E^*mut*^. Furthermore, IS3si universally showed synergistic inhibition of BaF3/G250E^*mut*^ and BaF3/F317L^*mut*^ when applied in combination with dasatinib (Fig. 3b, d). At last, the BaF3/G250E^*mut*^ cells showed resistance to nilotinib but the resistance was reversed when the cell line was treated with IS3si alone or a combination of IS3si and nilotinib (Fig. 3b). Of interest, T315I *BCR-ABL1* mutant (BaF3/T315I^*mut*^) cells also showed significant reduction in cell viability after treatment with either IS3si alone (to 36.45 ± 1.98%) or IS3si in combination with nilotinib (to 39.90 ± 2.82%) or dasatinib (to 29.23 ± 3.67%) (Fig. 3c). These data suggest that blockade of IS3 can inhibit the growth of both TKI-sensitive and TKI-resistant CML cells. The results of CrkL assay confirmed that IS3 blockade-induced CML cell killing effect is mediated through *BCR-ABL1* inhibition both in wild-type and mutant type CML cell lines including BaF3/T315I^*mut*^ cells (Supplementary Fig. 7). BaF3/T315I^*mut*^ cells especially did not show any decrease in the pCrkL/CrkL ratio after IM treatment. However, a significant reduction in the pCrkL/CrkL ratio was observed when these cells were treated with either IS3si alone or in combination with IM.

### Influence of IS3si on proliferation of the human K562/T315I^*mut*^ cell line

**Fig. 4:**
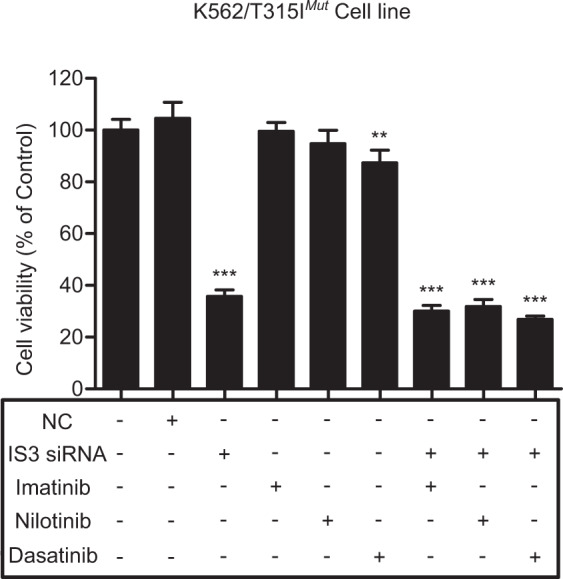
Results of cell viability assay using K562/T315I^*mut*^ cell line. Cell viability (%) was calculated as (absorbance of the treated wells−absorbance of the blank wells)/(absorbance of the control wells−absorbance of the blank wells) at 72 h time point. Cell viability was determined for untreated control, negative control (NC) treated with non-silencing siRNA, IS3si-treated, IM-, nilotinib-, or dasatinib-treated, and combination of IS3si plus IM-, nilotinib-, or dasatinib-treated cells. Statistical analysis was performed using Student’s t test with equal variance at 72 h. ***p < 0.001, **p < 0.01

We also confirmed the inhibitory effect of IS3si on K562/T315I^*mut*^ cell line that was developed using CRISPR/Cas9 system (Supplementary Fig. 8). Similar to the results observed in the BaF3/T315I^*mut*^ cells, treatment with either IS3si alone or in combination with IM significantly decreased the viability of K562/T315I^*mut*^ cells (Fig. 4). K562/T315I^*mut*^ cells were resistant to IM or nilotinib monotherapy, but the sensitivity to IM or nilotinib was reversed when these agents were combined with IS3 siRNA.

### IS3si suppresses colony-forming capacity of murine CML progenitor cells in vitro

**Fig. 5:**
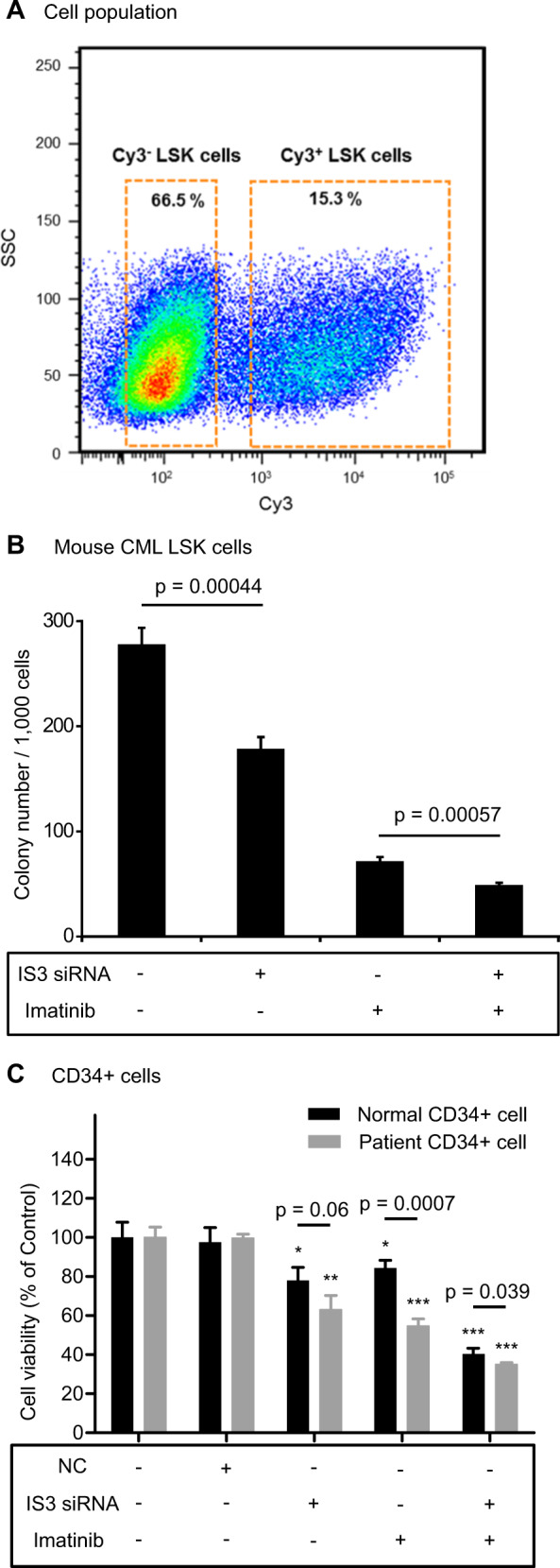
*HMGCLL1*-IS3 knockdown suppresses proliferation of murine and human CML stem cells in vitro. **a** The plot shows the Cy3-positive (IS3si transfected) Lineage^−^Sca1^+^cKit^+^ (LSK) cells and Cy3-negative (non-transfected) LSK cells population sorted by flow cytometry. SSC = side scatter. **b** The plot shows the effect of IS3si on murine CML LSK cells. Colony number per 1000 cells (*Y* axis) was counted after 7 days. Cy3-positive and Cy3-negative murine CML stem cells with or without IM (1 µm) were co-cultured with OP-9 stromal cells in vitro for 72 h. Colony-forming capacity of murine CML stem cells after treatment with IM was then determined. **c** The plot shows the effect of IS3si on human CD34^+^ cells isolated from clinical samples of patients with CML (CD34^+^/CML) and normal healthy individuals (CD34^+^/normal). Cell viability relative to control cells (*Y* axis) was measured at 192 h as average ± standard deviation (SD) from at least three independent wells. Cell viability (%) was calculated as follows: (absorbance of the treated wells−absorbance of the blank wells)/(absorbance of the control wells−absorbance of the blank wells). Examination of cell viability was performed for untreated control, negative control (NC) treated with non-silencing siRNA, IS3si-treated, IM-treated, and combination treatment with IS3si and IM-treated cells. Statistical analysis was performed using Student’s *t* test with equal variance. Asterisk marked *p* values were calculated by comparing with control fraction in each CD34^+^/normal or CD34^+^/CML, respectively. ***p  < 0.001, **p < 0.01, *p < 0.05

We used an established protocol to isolate Lineage^−^Sca1^+^cKit^+^ (LSK) cells from tetracycline-inducible CML-affected mice by cell sorting. These LSK cells were transfected with Cy3-labeled (fluorescence-marked) IS3si. Cy3-positive and Cy3-negative cells were then purified and assessed for their colony-forming capacity (Fig. 5a). Compared with Cy3-negative LSK cells, colony-forming capacity of Cy3-positive LSK cells was attenuated with or without IM. Thus, IS3 blockade decreases the colony-forming capacity of murine CML HPCs in vitro and may inhibit the repopulation capacity of murine CML-HPCs (Fig. 5b).

### Effects of IS3si on CD34^+^ cells from CML patients and normal healthy individuals-derived primary cells

Next, we investigated the effect of IS3 blockade on growth of primary CML cells isolated from patient samples. CD34^+^ fractions were isolated from samples of two CML patients (CD34^+^/CML) and one normal healthy donor (CD34^+^/Normal) and treated with IS3si with or without IM. Although marked cell growth inhibition was seen in both CD34^+^/CML and CD34^+^/Normal cells after IS3si treatment, there was a trend of greater inhibition of cell growth in the CD34^+^/CML fraction compared with the CD34^+^/Normal fraction (*p* *=* 0.060). IM therapy significantly decreased the growth of CD34^+^/CML fraction compared with the CD34^+^/Normal fraction (*p* < 0.001; Fig. 5c).

In addition to inhibiting *BCR-ABL1*, IM inhibits c-kit (KIT) and platelet-derived growth factor receptor, a prominent factor in normal hematopoietic stem cell development [29–32]. When IS3si was given in combination with IM, cell growth was further inhibited in CD34^+^/CML cells compared with CD34^+^/Normal cells (*p* *=* 0.039; Fig. 5c). This finding suggests that IS3 blockade could selectively suppress the CML-HPCs fraction (i.e., the CD34^+^ fraction in CML patients).

### RNA-seq reveals a mechanism related to IS3 isoform-mediated inhibition of CML cells

**Fig. 6:**
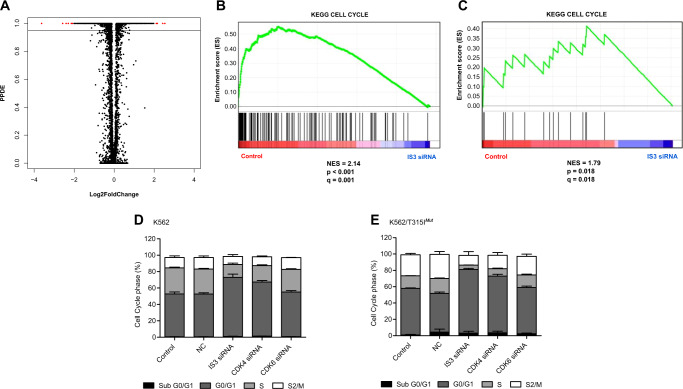
RNA sequencing results show that *HMGCLL1* blockade using IS3 siRNA can downregulate cell cycle mediated pathway. **a** The plot shows differentially expressed genes between control and IS3si-treated groups. The Y axis represents posterior probability that a gene/transcript is differentially expressed (PPDE). The *X* axis indicates the log2 Fold Change (FC) compared with the control. Black dots represent each gene excluding those with low read counts and normalized UQ < 100. Red dots indicate significantly different genes with PPDE = 1 and |log2FC| > 2. The black horizontal line indicates PPDE = 0.95. **b** The plot shows enrichment score plot by GSEA compared with control and IS3 blockade. Enrichment analysis was performed by GSEA using 12 134 genes selected following the criterion of UQ normalized mean read count value ≥ 100 for both groups: control and IS3si-treated. The cell cycle pathway was highly enriched by IS3 blockade as a schematic mechanism of CML (NES = 2.14 and FDR *q* val = 0.001 for KEGG CELL CYCLE). **c** Enrichment analysis was performed by GSEA using 404 genes selected based on expression below −1 fold or above 1 fold. As shown in the **b**, enrichment pathway analysis revealed that the cell cycle was only significantly in KEGG pathway sets (NES = 1.79 and *p* *=* 0.018 for KEGG CELL CYCLE). The vertical black line at the bottom indicates the members of gene set appearing in the ranked list of genes. **d** K562, and **e** K562/T315I^*mut*^. The plots show results of cell cycle analysis using a flow cytometer. Untreated control, NC, IS3, *CDK4*, and *CDK6* siRNA-treated K562 cells or K562/T315I^*mut*^ cells were fixed and stained with propidium iodide (PI) at the 48 h time point after transfection. Distribution of cells with respect to each cell cycle was analyzed as relative proportion of Annexin-V-positive/PI-negative cells. All data in the present cumulative bar graph are presented as average ± standard deviation from at least three independent experiments

To elucidate the downstream signaling pathways affected by IS3 blockade, we performed RNA-seq using K562 cell line that were treated with IS3si or vehicle control. In particular, the cyclin-dependent kinases, *CDK4* (*p* < 0.001, FC = −2.39) and *CDK6* (*p* < 0.001, FC = −2.13), expression was significantly decreased with IS3 siRNA treatment (Supplementary Table 10). *CDK4* and *CDK6* are frequently overexpressed and dysregulated in diverse types of cancers [33, 34]. The cyclin-dependent kinase *CDK6* is crucial gene associated with leukemia stem cells (LSCs) [35]. Using qPCR, we confirmed that *CDK4/6* gene expression was reduced after IS3si treatment (Supplemental Fig. 9). Gene set enrichment analysis (GSEA) showed that cell cycle gene set was the most significantly enriched pathway in the KEGG pathway sets (Familywise-error rate *p* *=* 0.001, Fig. 6a−c, Supplemental Table 11). Compared with vehicle control, suppression of K562 cells by IS3si promoted cell cycle arrest at G0/G1 phase and decreased S1 phase (*p* *=* 0.0017). IS3si-treated cells also displayed more profound cell cycle arrest at G0/G1 phase with decreased S1 phase compared with cells treated with *CDK4* or *CDK6* targeted siRNA. In K562/T315I^*mut*^ cells with high TKI resistance, increased cell cycle arrest at G0/G1 phase was observed by cell cycle assay (*p* < 0.001; Fig. 6d, e). These results are concordant with the results of IS3 blockade inhibition of cell growth. Thus, cell cycle arrest at G0/G1 phase can interfere with cell proliferation irrespective of T315I mutation in the *ABL1* kinase domain in vitro.

## Discussion

The present study demonstrates that the *HMGCLL1* genetic variant is a powerful predictive biomarker for intrinsic sensitivity to TKI therapy that correlates with DMR rate in CML patients. The following findings have been replicated in an independent cohort of CML patients with different ethnicities and their functional roles have been validated using siRNA-based inhibition in in vitro models: (1) The *HMGCLL1* genetic variant was a powerful predictive genetic biomarker for DMR with IM therapy, (2) Blockade of IS3 significantly reduced the viabilities of K562, CML-T1, BaF3/wild-type, and mutant BaF3 cell lines including the cell lines harboring *ABL1* kinase domain mutation such as T315I mutation, (3) IS3 blockade reduced colony-forming capacity of murine CML stem cells treated in vitro and selectively suppressed the CD34^+^ fractions isolated from CML patients compared with healthy control.

Of clinical relevance, this genetic biomarker can be used to identify the cases expected to have poor intrinsic sensitivity to IM therapy even prior to commencement of TKI therapy. Our data indicate that patients with TCGAATAC haplotype have a 35% and 34% lower chance of achieving DMR with IM therapy in the discovery set and validation set, respectively, and would be good candidates for frontline therapy with second-generation TKIs, if the goal of CML treatment is achieving treatment free remission, for which DMR is a prerequisite. As shown by GWAS, locus 6p12.1 that includes *HMGCLL1* had a strong correlation with DMR to IM therapy. TCGAATAC haplotype of *HMGCLL1* gene variant was also strongly associated with lower MMR rate with IM therapy (Supplementary Fig. 4 and Supplementary Table 2). Thus, this genetic biomarker can be utilized for prediction of both DMR and MMR.

*HMGCLL1*, also known as HMG-CoA Lyase Like 1, was first characterized as a novel lyase activity enzyme localized in extra-mitochondrial region [36, 37]. The role of *HMGCLL1* in susceptibility or resistance of cancer cells to chemotherapies has never been extensively investigated. Also, the biological relevance of *HMGCLL1* in HPCs has never been systemically elucidated. Accordingly, we have tested whether this gene is associated with susceptibility to CML development, and compared the frequency of this haplotype between CML and healthy control groups using the data previously published (Supplementary Table 5) [38].

Theoretically, *HMGCLL1* inhibition can be a potential therapeutic strategy to improve DMR in CML patients. In our study, blockade of *HMGCLL1* using siRNA significantly reduced the growth of *BCR-ABL1*^+^ cells, including K562, CML-T1, BaF3 wild-type, BaF3/T315I^*mut*^, and K562/T315I^*mut*^ cell lines. Blockade of IS3 could also decrease the HPC fraction isolated from CML patients and CML-affected mice (Fig. 5). Of surprise, IS3 blockade could overcome *ABL1* kinase domain mutations in BaF3 murine cell lines. Also, IS3 blockade could overcome the burden of T315I mutation in K562/T315I^*mut*^ cells generated by CRISPR/Cas9 system. These findings suggest that mechanistically IS3 blockade is not conflicting with ATP-pocket binding activity of TKIs, but functions through a different pathway. Thus, IS3 blockade could potentially be exploited to overcome TKI resistance in CML.

The results of the present study suggest that *HMGCLL1* blockade could potentially sensitize CML LSCs to TKI therapy and improve the DMR rate following this treatment. As a result, more patients may be eligible for TKI discontinuation, suggesting a possible role for pharmacological blockade of *HMGCLL1* gene or IS3 in treatment of CML.

Our RNA-seq data demonstrate that blockade of IS3 by IS3si in CML cells significantly decreases the expression levels of *CDK4* and *CDK6*, suggesting a role for *HMGCLL1* in cell cycle regulation in CML cells. This is strongly supported by consistent results of GSEA (in silico) and cell cycle analysis (in vitro) showing that the fraction of cell cycle arrest in G0/G1 phase is increased after IS3 blockade. Scheicher et al. have suggested that *CDK6* is a key regulator of hematopoietic and leukemic stem cell activation [39]. In addition, Ng et al. recently published a 17-gene LSC score (LSC17) signature that included *CDK6* as a marker to predict outcome in AML [35]. Given that the effect of *HMGCLL1* inhibition on *BCR-ABL1*^+^ cell growth was mediated by the *CDK4/6* pathway, *CDK4/6* inhibitors such as palbociclib should be further investigated as a potential therapeutic option for CML treatment (Supplementary Fig. 9). Further preclinical studies are needed to determine the clinical applicability of *HMGCLL1* or *CDK4/6* inhibition in CML to improve DMR rates and enhance LSCs eradication.

In summary, our results highlight that the *HMGCLL1* gene polymorphism is a predictive genetic biomarker for intrinsic sensitivity to IM therapy in CML and can be used to identify high risk patients that may fail to achieve DMR with IM therapy even prior to initiation of treatment.

## Supplementary information

Supplementary Information
