# Supplementary material for: HMGCLL1 is a predictive biomarker for deep molecular response to imatinib therapy in chronic myeloid leukemia
Source: Leukemia. 2018 Dec 16;33(6):1439–50. doi: 10.1038/s41375-018-0321-8 (PMC6756062; doi:10.1038/s41375-018-0321-8)
Supplement: Supplementary file 1 — Supplementary Information [file 41375_2018_321_MOESM1_ESM.docx]

**SUPPLEMENTAL INFORMATION**

**Supplemental Methods**

**Description of participants and genotyping**

A total of 202 Korean CML patients and 272 European CML patients were recruited for use as a discovery set and validation set, respectively. The study was approved by the Institutional Research Board at the Samsung Medical Center, Seoul, Korea.

A total of 906 530 single nucleotide polymorphisms (SNPs) were genotyped using the Affymetrix Genome-Wide Human SNP Array 6.0 (Affymetrix, Santa Clara, CA, USA). Yields of pure, double-stranded genomic DNA were determined using the QIAamp DNA blood Maxi Kit (Qiagen, Valencia, CA, USA). Peripheral blood samples were taken during the course of therapy, usually when or after achieving complete cytogenetic response or achieving 2-log reduction in the *BCR-ABL1* mRNA level and after informed consent was obtained in accordance with the Declaration of Helsinki. Samples were normalized to 50 ng/μL, and the normalized genomic DNA (5 μL) from each sample was used as a template for Affymetrix Version 6.0 assays. Genotyping reactions were performed using Affymetrix Genome-Wide Human SNP Nsp/Sty, Version 6.0 kit reagents and protocols. Genotypes were called using the Birdseed algorithm of the Affymetrix Genotyping Console Version 3.0.2 [[1](#_ENREF_1_1)].

**Definition of response receiving imatinib therapy**

A complete molecular response was defined as no Philadelphia chromosome-positive (Ph^+^) cells in metaphase. Major molecular response was defined as a reduction of at least 3-log in the *BCR-ABL1* mRNA level, and deep molecular response was defined as a reduction of at least 4-log or undetectable transcript level of the *BCR-ABL1* by Quantitative *BCR-ABL1* mRNA PCR on peripheral blood. This was performed according to the manufacturer's instructions using ABI 7900 Thermal Cycler (Applied Biosystems, Foster City, CA, USA). Standardization procedure to international scale was conducted per recommendation. Sensitivity of the assay was up to 4.5 log reduction with minimum number of reference gene transcripts of 32 000 copies *ABL1*.

**Fine mapping to find causal functional variants associated with CML in *HMGCLL1***.

To identify functional variants among SNPs as predictive surrogates for DMR to IM therapy, 15 samples from each of the Korean and European ethnicity patients were chosen for targeted re-sequencing using the Ion Torrent Personal Genome Machine (PGM) (Life Technologies, San Francisco, CA, USA) based on PCR target enrichment and next-generation sequencing (AmpliSeq). Customized AmpliSeq primers of *HMGCLL1*, including a whole gene region of ±25kb, were designed using the Ion AmpliSeq Designer. Library construction was performed using the Ion AmpliSeq Library kit. IonXpress Barcodes were used for multiplexing with a 10ng DNA sample per pool. Clonal amplification was accomplished for constructed libraries by emulsion PCR (emPCR). Ion PGM enrichment was performed using enrichment beads. Final products were applied to Ion 318 Chip and sequenced on a PGM Sequencer. Sequencing data were analyzed using Torrent Suite Software 4.0.2 and aligned with reference genome hg19 using the Torrent Mapping Alignment Program (TMAP). Variant detection was performed using VariantCaller v4.0-r76860 (Torrent server plugin). Variant annotation was conducted using ANNOVAR for RefGene, dbSNP 137, and population frequency from the 1000 genome and ESP 6500 [[2](#_ENREF_1_2)].

**Genotyping and quantitative polymerase chain reaction (qPCR) for *HMGCLL1* and isoform mRNA expression for expression quantitative trait loci (eQTL) analysis**.

To evaluate whether SNPs identified in the genome-wide association study (GWAS) were functional, we determined variation at the gene expression level associated with the genotype known as eQTL [[3](#_ENREF_1_3)]. A total of 110 healthy donors of Korean ethnicity who had no history of blood disorder or abnormal blood counts were used for eQTL analysis. Polymorphonuclear cells (PMNs) were isolated from peripheral blood using Ficoll density gradient method. Genotyping was performed on MassARRAY iPLEX system (Agena Bioscience, San Diego, CA, USA). Based on results obtained from fine mapping using AmpliSeq, 8 SNPs (rs10948926, rs10948927, rs9370435, rs4546489, rs4275061, rs9475323, rs9475327, and rs9296791) were selected for genotyping. MassARRAY Assay Design 3.1 software was used to design multiplex primers for each SNP.

Isoform specific qPCR primers were designed to capture exon-exon junctions and to quantify gene expression of each isoform (**Supplementary Table 12**). Target sites of isoform specific primers for amplification of transcripts are shown in **Supplementary Fig. 5a**. Amplification was done on an ABI 7900 HT Real-Time PCR System. For qPCR, total cDNA was amplified using SYBR Select Master Mix (Life Technologies, Austin, TX, USA). After 45 cycles at 95 °C for 5 sec and 60 °C for 1 min, dissociation curve analysis for ΔCt was analyzed using SDS 2.4 and ABI RQ manager software. For quantification, RNA transcript expression was normalized by determining the ratio between expression level of *HMGCLL1* and that of *GAPDH* gene.

**Cell lines for *in vitro* assay**.

Cell lines used in this study included K562 (obtained from the American Type Culture Collection), CML-T1 (obtained from Deutsche Sammlung von Mikroorganismen und Zellkulturen GmbH), and BaF3 cells (including wild type and mutant type cell lines: BaF3/WT, BaF3/G250E*^mut^*, BaF3/T315I*^mut^*, and BaF3/F317L*^mut^* provided by Dr. Shinya Kimura at Saga University School of Medicine). All cell lines were cultured in RPMI-1640 media (Invitrogen, Carlsbad, CA, USA) or DMEM (Invitrogen, Carlsbad, CA, USA) supplemented with 10 % fetal bovine serum (FBS; Invitrogen, Carlsbad, CA, USA), 100 units/mL penicillin, and 100 µg/mL streptomycin (Invitrogen, Carlsbad, CA, USA).

**Hematopoietic progenitor cells isolated from primary samples of CML patients**.

Bone marrow (BM) samples from CML patients collected at the time of initial diagnosis of CML were obtained and processed. Primary normal BM CD34^+^ cells (PCS-800-012) were obtained from ATCC. Total cells were washed and resuspended in SFEM II medium (STEMCELL Technologies, Vancouver, BC, Canada) at density of 1×10^6^ cells/ml and stained with 5 μg/ml Hoechst 33342 (Sigma-Aldrich) for 90 minutes at 37 °C. Cells were incubated with FITC-labeled anti-CD34 Ab (BD Bioscience Pharmingen) for 30 min at 4 °C after Hoechst staining prior to flow cytometry. CD34^+^ cells were isolated by positive selection according to previously described methods [[4-6](#_ENREF_1_4)]. Purity of CD34^+^ cells reproducibly exceeded 98% as determined by flow-cytometric analysis using FACSAria III Cell-Sorting System (BD Biosciences, San Jose, CA, USA).

**siRNA transfection and drug administration**.

Primers and siRNAs were chemically synthesized (GE Healthcare Dharmacon, Lafayette, CO, USA and Bioneer, South Korea) (**supplementary Table 12**). Non-silencing siRNA was also synthesized and used as negative control (NC). Chemically synthesized siRNAs at concentrations of 100 pmol per 2×10^5^ cells were transfected into cells by electroporation method using Neon Transfection System (Invitrogen). Transfection rate at 24 hours was evaluated by fluorescence-marked cells with Cy3 and quantified using FACSVerse. Cells were then resuspended in medium containing 20% FBS and further incubated at 37 °C. For tests using a combination of siRNA and TKIs, cells were treated with TKIs including imatinib (IM; Gleevec, Novartis, Basel, Switzerland), Nilotinib (Tasigna, Selleckchem, Houston, TX, USA), and Dasatinib (Sprycel, Selleckchem, Houston, TX, USA) for 0 to 72 h. Concentrations of these drugs used *in vitro* treatment were 500 nM for IM, 70 nM for nilotinib, and 10 nM for dasatinib. Each chosen concentration has been used based on human pharmacokinetic parameters and preliminary evidence in cell line experiments.

**Monitoring cell growth at each time point**.

K562, CML-T1, and BaF3 cells were plated into 96-well plates in triplicates at a density of 3×10^3^ cells/well. Cell viability was assessed at 0, 24, 48, and 72 h after transfection and/or exposure to TKIs based on the WST-8 assay (Dojindo Laboratories, Kumamoto, Japan) [[7](#_ENREF_1_7)]. After incubating with the WST-8 reagent at 37 °C for 4 hours, absorbance was measured at wavelength of 450 nm using xMark Microplate Absorbance Spectrophotometer (Bio-Rad Laboratories, Hercules, CA, USA). Isolated CD34^+^ cells were plated into 96-well plates (500 cells/well) with 100 mL of SFEM II medium containing RealTime-Glo MT Cell Viability Assay reagents (Promega, Madison, WI, USA). Cell viability was assessed every 24 h for 8 days using a GloMax-Multi Detection System (Luminometer; Promega, Madison, USA) [[8](#_ENREF_1_8), [9](#_ENREF_1_9)]. The percentage of viable cells in comparison with non-treated control cells was determined. We performed more than three independent experiments with at least triplicates to ensure accuracy.

**phospho-CrkL (pCrkL) assay and cell cycle analysis**.

For measurement of pCrkL/CrkL ratio, cells transfected with IS3-targeted small interfering RNA (IS3si) and/or TKIs were assayed using pCrkL (Tyr207) Colorimetric Cell-Based Enzyme Linked Immunosorbent Assay (ELISA) Kit (Aviva Systems Biology, San Diego, CA, USA). Cell cycle analysis was performed using standard protocols. Control, NC, IS3, *CDK4*, and *CDK6* siRNA treated cells were fixed, stained with propidium iodide (PI), and then analyzed using FACSVerse flow cytometry (BD Biosciences, San Jose, CA, USA). Distribution of cells in different phases of the cell cycle was analyzed using CellQuest Pro software (BD Biosciences).

**Developing K562/T315I*^mut^* drug-resistant mode**l.

To generate an isogenic K562 cell line containing a TKI resistant mutation, the CRISPR/Cas9 system was employed as a genome editing method, using the Cas9 RNA guided DNA endonuclease [[10](#_ENREF_1_10), [11](#_ENREF_1_11)]. As a guide RNA to target the ABL kinase domain within the *BCR-ABL1* fusion gene, we designed a single guide RNA (sgRNA) and two compliment oligo primers. Cloning of sgRNA was performed using the Guide-it CRISPR/Cas9 System (Clontech Laboratories, Palo Alto, CA, USA) according to the manufacturer’s instruction. Introduction of the T315I mutation (c.944C>T) into the K562 cell line (K562/T315I*^mut^*) was single-clone selected and validated using capillary sequencing (3130XL Genetic Analyzer; Applied Biosystems, Foster City, CA, USA).

**CML murine model***.*

*Scl/Tal1-tTA* (#006209) and *TRE-BCR-ABL1* (#006202) transgenic mice were obtained from the Jackson Laboratory (Bar Harbor, ME, USA). We backcrossed these strains into C57BL/6 genetic background (F5) [[12](#_ENREF_1_12)]. *Scl/Tal1-tTA* and *TRE-BCR-ABL1* transgenic mice were then interbred to generate *Scl/Tal1-tTA* x *TRE-BCR-ABL1* double transgenic mice. These animals were maintained in cages supplied with drinking water containing 20 mg/L doxycycline (DOX; Sigma-Aldrich, Saint Louis, MO, USA). At 5 weeks after birth, expression of *BCR-ABL1* oncogene was induced by replacing DOX-containing drinking water with normal drinking water. CML-like disease developed in these double transgenic mutants at about five weeks after DOX withdrawal [[12-15](#_ENREF_1_12)]. We examined three independent experiments, used three CML-affected mice in the 1^st^ experiment, four in the 2^nd^ and three in the 3^rd^. All animal care was in accordance with the guidelines for animal and recombinant DNA experiments of Hiroshima University.

**CML stem cells isolated from murine CML model**.

To evaluate colony-forming capacity of murine CML stem cells co-cultured with OP-9 stromal cells *in vitro*, Lineage^-^Sca1^+^cKit^+^ (LSK) cells were isolated from tetracycline-inducible CML-affected mice as described previously [[12](#_ENREF_1_12), [13](#_ENREF_1_13)]. BM cells were isolated from two hind limbs of each tetracycline-inducible CML-affected mouse at 5 weeks after withdrawal of DOX. These cells were stained with anti-Sca-1 (E13-161.7)-PE, anti-CD4 (L3T4)-FITC, anti-CD8 (53-6.7)-FITC, anti-B220 (RA3-6B2)-FITC, anti-TER119 (Ly-76)-FITC, anti-Gr-1 (RB6-8C5)-FITC, anti-Mac1 (M1/70)-FITC (all from BD Bioscience Pharmingen, San Diego, CA, USA), and anti-cKit (ACK2)-APC (eBioscience, San Diego, CA, USA) antibodies. They were then sorted by flow cytometry (BD FACSAria III) to isolate cell fractions containing LSK cells.

**RNA interference in murine CML stem cells**.

LSK cells isolated from BM of leukemic mice were transfected with Cy3-labeled (fluorescence-marked) IS3si using the Lipofectamine RNAiMAX reagent (Life Technologies) in S-clone SF-O3 medium (Sanko Junyaku, Tokyo, Japan) without cytokines or penicillin/streptomycin for three hours under hypoxic conditions (3% O_2_). These cells were then cultured for 3 days under hypoxic conditions in SF-O3 medium containing BSA, TPO (100 nM), SCF (100 nM), and penicillin/streptomycin. Cy3-negative and Cy3-positive cells were then sorted using BD FACSAria III.

**Colony-forming assays in murine CML stem cells**.

To determine colony-forming capacity, cells were co-cultured on OP-9 stromal cells for 72 hours under hypoxic (3% O_2_) conditions. Cells were then washed with PBS and transferred into a semi-solid methylcellulose medium containing cytokines SCF, IL-3, IL-6, and erythropoietin (MethoCult GF M3434; Stemcell Technologies, Vancouver, Canada) under hypoxic conditions. Colonies (CFU-GEMM) were counted 7 days later under a microscope [[12](#_ENREF_1_12), [13](#_ENREF_1_13)]. Each colony assay were performed with triplicate.

**RNA sequencing to define molecular pathway of *HMGCLL1* blockade in *BCR-ABL1*^+^** **cells**.

To explore the functional pathway of *HMGCLL1* blockade in *BCR-ABL1*^+^ cells, RNA sequencing was performed. Cy3-labeled (fluorescence-marked) IS3si was transfected into cells by electroporation. These cells were then resuspended in DMEM medium containing 20% FBS and further incubated at 37 °C with 5 % CO_2_ for 24h. After incubation, sorting of transfected cells for gene-specific Cy3 fluorescence with an excitation wavelength of 546 nm (emission 575–640 nm) was carried out on a BD FACSAria III. Total RNA was extracted from each sample (Control and siRNA-treated groups) using TRIzol reagent (Invitrogen, Carlsbad, CA, USA). Each sample was prepared in triplicate for differentially expressed gene (DEG) analysis. RNA-seq library was prepared using the TruSeq RNA Sample Prep Kit v2 (Illumina, San Diego, CA, USA) to capture coding transcriptome without strand information. Sequencing library was prepared by random fragmentation of total RNA with random hexamer followed by 5’ and 3’ adapter ligation. Non-stranded RNA-seq was performed on HiSeq 2500 (Illumina, San Diego, CA, USA) with 2×101 bp paired-end sequencing. Adapter trimming was performed using Trimomatic-0.33 before read alignment with reference genome (Ensembl, GRCh37) [[16](#_ENREF_1_16)]. Trimming threshold was determined by ILLUMINACLIP to cut Illumina-specific adapter sequences, allowing maximally two mismatches with 30 for clipping paired-end reads score. Adapter sequences were used for TruSeq3-PE adapter generated for TruSeq sequencing library construction. Trimmed paired reads were aligned with human reference genome release version GRCh37 with Ensembl General Transfer Format (GTF) gene annotation using STAR 2.4.2a [[17](#_ENREF_1_17)]. Ensembl gene annotation files were downloaded from Ensembl release 75 through Illumina iGenomes collection of reference sequences and annotation files. Ensembl release 75 comprised of 20 805 coding genes, 9 096 short non-coding genes, 13 870 long non-coding genes, and 14 181 pseudogenes. RNA-seq data quality was evaluated using RSeQC v2.6.4 [[18](#_ENREF_1_18)]. To calculate expression level, raw read counts were generated using RSEM v1.3.0 [[19](#_ENREF_1_19)]. We examined calculated expression levels of 57 773 transcribed genes between control and IS3si treated groups. Total number of reads within each sample was computed with Upper-Quantile (UQ) normalization. DEG analysis between Control and IS3si treated groups was performed using EBSeq [[20](#_ENREF_1_20)]. DEG analysis was then performed for 32 632 genes with non-zero raw read counts. Average expression level was calculated from triplicated samples within each group. As a DEG results, we also found that 4 642 genes had posterior probability of 1 to be differentially expressed (PPDE) between the two groups. GSEA was used for pathway enrichment analysis for DEGs obtained from RNA-seq data [[21](#_ENREF_1_21)]. Gene sets were defined by KEGG pathway database downloaded from the Broad ftp website [[22](#_ENREF_1_22)]. For examples, gene set file c2.cp.kegg.v6.0.symbols contained C2 gene sets exported from the Molecular Signature Database (MSigDB) [[23](#_ENREF_1_23)].

**Supplementary References**

1. Kim DH, Lee ST, Won HH, Kim S, Kim MJ, Kim HJ, et al. A genome-wide association study identifies novel loci associated with susceptibility to chronic myeloid leukemia. Blood. 2011;117:6906-11.

2. Wang K, Li M, Hakonarson H. ANNOVAR: functional annotation of genetic variants from high-throughput sequencing data. Nucleic acids research. 2010;38:e164.

3. Westra HJ, Peters MJ, Esko T, Yaghootkar H, Schurmann C, Kettunen J, et al. Systematic identification of trans eQTLs as putative drivers of known disease associations. Nature genetics. 2013;45:1238-43.

4. Goodell MA, Rosenzweig M, Kim H, Marks DF, DeMaria M, Paradis G, et al. Dye efflux studies suggest that hematopoietic stem cells expressing low or undetectable levels of CD34 antigen exist in multiple species. Nature medicine. 1997;3:1337-45.

5. Uchida N, Fujisaki T, Eaves AC, Eaves CJ. Transplantable hematopoietic stem cells in human fetal liver have a CD34(+) side population (SP)phenotype. The Journal of clinical investigation. 2001;108:1071-7.

6. Ramos CA, Venezia TA, Camargo FA, Goodell MA. Techniques for the study of adult stem cells: be fruitful and multiply. BioTechniques. 2003;34:572-8, 80-4, 86-91.

7. Tominaga H, Ishiyama M, Ohseto F, Sasamoto K, Hamamoto T, Suzuki K, et al. A water-soluble tetrazolium salt useful for colorimetric cell viability assay. Anal Commun. 1999;36:47-50.

8. Duellman SJ, Zhou W, Meisenheimer P, Vidugiris G, Cali JJ, Gautam P, et al. Bioluminescent, Nonlytic, Real-Time Cell Viability Assay and Use in Inhibitor Screening. Assay and drug development technologies. 2015;13:456-65.

9. Hata AN, Niederst MJ, Archibald HL, Gomez-Caraballo M, Siddiqui FM, Mulvey HE, et al. Tumor cells can follow distinct evolutionary paths to become resistant to epidermal growth factor receptor inhibition. Nature medicine. 2016;22:262-9.

10. Hsu PD, Scott DA, Weinstein JA, Ran FA, Konermann S, Agarwala V, et al. DNA targeting specificity of RNA-guided Cas9 nucleases. Nature biotechnology. 2013;31:827-32.

11. Mali P, Aach J, Stranges PB, Esvelt KM, Moosburner M, Kosuri S, et al. CAS9 transcriptional activators for target specificity screening and paired nickases for cooperative genome engineering. Nature biotechnology. 2013;31:833-8.

12. Naka K, Jomen Y, Ishihara K, Kim J, Ishimoto T, Bae EJ, et al. Dipeptide species regulate p38MAPK-Smad3 signalling to maintain chronic myelogenous leukaemia stem cells. Nature communications. 2015;6:8039.

13. Naka K, Ishihara K, Jomen Y, Jin CH, Kim DH, Gu YK, et al. Novel oral transforming growth factor-beta signaling inhibitor EW-7197 eradicates CML-initiating cells. Cancer science. 2016;107:140-8.

14. Koschmieder S, Gottgens B, Zhang P, Iwasaki-Arai J, Akashi K, Kutok JL, et al. Inducible chronic phase of myeloid leukemia with expansion of hematopoietic stem cells in a transgenic model of BCR-ABL leukemogenesis. Blood. 2005;105:324-34.

15. Huettner CS, Zhang P, Van Etten RA, Tenen DG. Reversibility of acute B-cell leukaemia induced by BCR-ABL1. Nature genetics. 2000;24:57-60.

16. Bolger AM, Lohse M, Usadel B. Trimmomatic: a flexible trimmer for Illumina sequence data. Bioinformatics. 2014;30:2114-20.

17. Dobin A, Davis CA, Schlesinger F, Drenkow J, Zaleski C, Jha S, et al. STAR: ultrafast universal RNA-seq aligner. Bioinformatics. 2013;29:15-21.

18. Wang L, Wang S, Li W. RSeQC: quality control of RNA-seq experiments. Bioinformatics. 2012;28:2184-5.

19. Li B, Dewey CN. RSEM: accurate transcript quantification from RNA-Seq data with or without a reference genome. BMC Bioinformatics. 2011;12:323.

20. Leng N, Dawson JA, Thomson JA, Ruotti V, Rissman AI, Smits BM, et al. EBSeq: an empirical Bayes hierarchical model for inference in RNA-seq experiments. Bioinformatics. 2013;29:1035-43.

21. Subramanian A, Tamayo P, Mootha VK, Mukherjee S, Ebert BL, Gillette MA, et al. Gene set enrichment analysis: a knowledge-based approach for interpreting genome-wide expression profiles. Proceedings of the National Academy of Sciences of the United States of America. 2005;102:15545-50.

22. Kanehisa M, Goto S. KEGG: kyoto encyclopedia of genes and genomes. Nucleic acids research. 2000;28:27-30.

23. Liberzon A, Subramanian A, Pinchback R, Thorvaldsdottir H, Tamayo P, Mesirov JP. Molecular signatures database (MSigDB) 3.0. Bioinformatics. 2011;27:1739-40.

| **Supplementary Table and Figure List** | |
| --- | --- |
| Table S1 | List of SNPs in 6p12.1 and 16q23.3 associated with deep molecular response achievement following imatinib therapy in the discovery set (*p* < 1.0E-04) |
| Table S2 | Association of eight SNPs with major molecular response and complete cytogenetic response which has been validated as a marker for deep molecular response following imatinib therapy. |
| Table S3 | List of SNPs (n=160) associated with complete cytogenetic response achievement following imatinib therapy in the discovery set (*p* < 1.0E-04) |
| Table S4 | List of SNPs (n=135) associated with major molecular response achievement following imatinib therapy in the discovery set (*p* < 1.0E-04) |
| Table S5 | No association of eight SNPs with chronic myeloid leukemia susceptibility which has been validated as a marker for deep molecular response following imatinib therapy. |
| Table S6 | The follow-up duration following imatinib therapy of selected patients in fine-mapping study |
| Table S7 | The types of *HMGCLL1* alternative isoform based on public databases (NCBI and Ensembl) |
| Table S8 | The expression level of splicing transcript type of *HMGCLL1* is associated with TCGAATAC haplotype identified by expression quantitative trait loci analysis. |
| Table S9 | Results of cell viability assay using various siRNAs to interfere specific isoforms in K562 cell line at 72h. |
| Table S10 | RNA sequencing results show that *HMGCLL1* blockade using IS3 siRNA can downregulate cell cycle mediated genes |
| Table S11 | Detailed enrichment results by GSEA compared to control and IS3 blockade |
| Table S12 | Primer and siRNA sequences used for in vitro assay |
| Figure S1 | Regional plot of candidate regions in the discovery set |
| Figure S2 | LD block patterns within the validated gene, *HMGCLL1*, in East Asian and European population |
| Figure S3 | Cumulative incidence plot of deep molecular response (DMR) in only chronic phase (CP) patients in the discovery and validation sets, respectively |
| Figure S4 | Cumulative incidence plot of major molecular response (MMR) in the discovery and validation sets, respectively |
| Figure S5 | Splicing transcript types of *HMGCLL1* and eQTL results associated with TCGAATAC haplotype |
| Figure S6 | Splicing transcript types of *HMGCLL1* and targeted siRNA binding sites to interfere each isoform |
| Figure S7 | Phospho-CrkL (pCrkL)/CrkL ratio assessed as *BCR-ABL1* activity in K562, BaF3/WT, and BaF3/T315I cell lines |
| Figure S8 | Confirmation of acquired heterozygous T315I mutation in K562 cell line using genome editing technology based on CRISPR/Cas9 system |
| Figure S9 | Decreased expression level of *CDK4* and *CDK6* after IS3si treatment was confirmed using qPCR in K562 and LAMA84 cell lines |

**Table S1. List of SNPs in 6p12.1 and 16q23.3 associated with deep molecular response achievement following imatinib therapy in the discovery set (*p* < 1.0E-04)**

| **Chromosome** | **SNP** | **Position*** | **Nearby Gene** | **Minor allele** | **Major allele** | **MAF in Response** | **MAF in Non-response** | ***p* value** | **HR (CI, 95%)** |
| --- | --- | --- | --- | --- | --- | --- | --- | --- | --- |
| 6 | rs6459081 | 55352586 | *HMGCLL1* | A | T | 0.52 | 0.43 | 2.25E-05 | 1.86 (1.39-2.47) |
| 6 | rs12528088 | 55355591 | *HMGCLL1* | A | T | 0.52 | 0.43 | 2.25E-05 | 1.86 (1.39-2.47) |
| 6 | rs9382497 | 55361392 | *HMGCLL1* | G | C | 0.38 | 0.45 | 7.47E-05 | 0.54 (0.40-0.73) |
| 6 | rs9475319 | 55361878 | *HMGCLL1* | G | A | 0.52 | 0.43 | 2.25E-05 | 1.86 (1.39-2.47) |
| 6 | rs10948926 | 55366347 | *HMGCLL1* | T | G | 0.38 | 0.45 | 7.47E-05 | 0.54 (0.40-0.73) |
| 6 | rs10948927 | 55366392 | *HMGCLL1* | C | T | 0.38 | 0.45 | 7.47E-05 | 0.54 (0.40-0.73) |
| 6 | rs10948931 | 55369699 | *HMGCLL1* | A | G | 0.37 | 0.45 | 3.54E-05 | 0.52 (0.38-0.71) |
| 6 | rs9370435 | 55369916 | *HMGCLL1* | G | C | 0.37 | 0.45 | 3.48E-05 | 0.52 (0.38-0.71) |
| 6 | rs4546489 | 55370236 | *HMGCLL1* | A | T | 0.37 | 0.45 | 4.21E-05 | 0.53 (0.39-0.71) |
| 6 | rs4275061 | 55371577 | *HMGCLL1* | A | G | 0.38 | 0.45 | 7.47E-05 | 0.54 (0.40-0.73) |
| 6 | rs4626423 | 55371663 | *HMGCLL1* | A | C | 0.37 | 0.45 | 5.36E-05 | 0.53 (0.39-0.72) |
| 6 | rs9475323 | 55371939 | *HMGCLL1* | T | C | 0.38 | 0.45 | 7.47E-05 | 0.54 (0.40-0.73) |
| 6 | rs9475327 | 55372270 | *HMGCLL1* | A | G | 0.38 | 0.45 | 7.61E-05 | 0.54 (0.40-0.73) |
| 6 | rs9475328 | 55372418 | *HMGCLL1* | A | G | 0.38 | 0.45 | 7.47E-05 | 0.54 (0.40-0.73) |
| 6 | rs7751266 | 55373299 | *HMGCLL1* | A | G | 0.38 | 0.45 | 7.47E-05 | 0.54 (0.40-0.73) |
| 6 | rs9296791 | 55376167 | *HMGCLL1* | C | T | 0.38 | 0.45 | 7.47E-05 | 0.54 (0.40-0.73) |
| 6 | rs4278020 | 55376330 | *HMGCLL1* | T | C | 0.36 | 0.46 | 2.51E-05 | 0.51 (0.37-0.69) |
| 6 | rs13206666 | 55378633 | *HMGCLL1* | C | T | 0.38 | 0.45 | 7.47E-05 | 0.54 (0.40-0.73) |
| 6 | rs7760065 | 55387333 | *HMGCLL1* | A | T | 0.38 | 0.46 | 7.12E-05 | 0.52 (0.38-0.72) |
| 6 | rs7740208 | 55387415 | *HMGCLL1* | C | T | 0.38 | 0.47 | 3.86E-05 | 0.52 (0.38-0.71) |
| 6 | rs7452170 | 55390875 | *HMGCLL1* | T | C | 0.37 | 0.45 | 5.72E-05 | 0.53 (0.39-0.72) |
| 6 | rs7744273 | 55391815 | *HMGCLL1* | A | G | 0.38 | 0.47 | 3.94E-05 | 0.52 (0.38-0.71) |
| 16 | rs3784950 | 83734649 | *CDH13* | T | C | 0.27 | 0.18 | 6.00E-05 | 1.85 (1.37-2.51) |
| 16 | rs424280 | 83741244 | *CDH13* | T | C | 0.23 | 0.13 | 4.64E-06 | 2.09 (1.53-2.87) |
| 16 | rs375142 | 83747327 | *CDH13* | C | G | 0.16 | 0.09 | 1.45E-05 | 2.25 (1.56-3.25) |
| 16 | rs254326 | 83747850 | *CDH13* | T | C | 0.16 | 0.09 | 2.82E-05 | 2.21 (1.53-3.21) |
| 16 | rs254315 | 83756412 | *CDH13* | G | A | 0.17 | 0.09 | 5.68E-06 | 2.31 (1.61-3.33) |
| 16 | rs374476 | 83757149 | *CDH13* | C | T | 0.16 | 0.08 | 1.86E-05 | 2.24 (1.55-3.23) |

MAF, minor allele frequency; HR, hazard ratio; CI, confidence interval; SNP, single nucleotide polymorphism.

* Physical position based on human reference genome build hg 19 (GRCh37).

**Table S2. Association of eight SNPs with major molecular response and complete cytogenetic response which has been validated as a marker for deep molecular response following imatinib therapy.**

|  |  |  |  | **Deep molecular response** | | | | **Major molecular response** | | | | **Complete cytogenetic response** | | | | |
| --- | --- | --- | --- | --- | --- | --- | --- | --- | --- | --- | --- | --- | --- | --- | --- | --- |
| **Chr** | **SNP** | **Position†** | **Minor/Major**  **allele** | **MAF in**  **Response** | **MAF in**  **Non-response** | ***p* value** | **HR (CI, 95%)** | **MAF in**  **Response** | **MAF in**  **Non-response** | ***p* value** | **HR (CI, 95%)** | **MAF in**  **Response** | **MAF in**  **Non-response** | ***p* value** | **HR (CI, 95%)** |  |
| 6 | rs10948926 | 55366347 | T/G | 0.38 | 0.45 | 7.47E-05 | 0.54 (0.40-0.73) | 0.40 | 0.44 | 9.12.E-04 | 0.66 (0.52-0.84) | 0.42 | 0.40 | 0.048 | 0.797 (0.636-0.998) |  |
| 6 | rs10948927 | 55366392 | C/T | 0.38 | 0.45 | 7.47E-05 | 0.54 (0.40-0.73) | 0.40 | 0.44 | 9.12.E-04 | 0.66 (0.52-0.84) | 0.42 | 0.40 | 0.048 | 0.797 (0.636-0.998) |  |
| 6 | rs9370435 | 55369916 | G/C | 0.37 | 0.45 | 3.48E-05 | 0.52 (0.38-0.71) | 0.40 | 0.44 | 5.43.E-04 | 0.65 (0.50-0.83) | 0.42 | 0.40 | 0.036 | 0.785 (0.626-0.985) |  |
| 6 | rs4546489 | 55370236 | A/T | 0.37 | 0.45 | 4.21E-05 | 0.53 (0.39-0.71) | 0.40 | 0.43 | 6.17.E-04 | 0.65 (0.51-0.83) | 0.42 | 0.39 | 0.040 | 0.789 (0.629-0.989) |  |
| 6 | rs4275061 | 55371577 | A/G | 0.38 | 0.45 | 7.47E-05 | 0.54 (0.40-0.73) | 0.40 | 0.44 | 9.12.E-04 | 0.66 (0.52-0.84) | 0.42 | 0.40 | 0.048 | 0.797 (0.636-0.998) |  |
| 6 | rs9475323 | 55371939 | T/C | 0.38 | 0.45 | 7.47E-05 | 0.54 (0.40-0.73) | 0.40 | 0.44 | 9.12.E-04 | 0.66 (0.52-0.84) | 0.42 | 0.40 | 0.048 | 0.797 (0.636-0.998) |  |
| 6 | rs9475327 | 55372270 | A/G | 0.38 | 0.45 | 7.61E-05 | 0.54 (0.40-0.73) | 0.40 | 0.43 | 9.33.E-04 | 0.66 (0.52-0.84) | 0.42 | 0.39 | 0.050 | 0.798 (0.637-1.000) |  |
| 6 | rs9296791 | 55376167 | C/T | 0.38 | 0.45 | 7.47E-05 | 0.54 (0.40-0.73) | 0.40 | 0.44 | 9.12.E-04 | 0.66 (0.52-0.84) | 0.42 | 0.40 | 0.048 | 0.797 (0.636-0.998) |  |

HR, Hazard ratio; CI, confidence interval; MAF, minor allele frequency.

†Physical position based on human reference genome build hg 19 (GRCh37).

**Table S3. List of SNPs (n=160) associated with complete cytogenetic response achievement following imatinib therapy in the discovery set (*p* < 1.0E-04)**

| **Chromosome** | **SNP** | **Position*** | **Nearby Gene** | **Minor**  **allele** | **Major**  **allele** | **MAF**  **in Response** | **MAF**  **in Non-response** | ***p* value** | **HR (CI, 95%)** |
| --- | --- | --- | --- | --- | --- | --- | --- | --- | --- |
| 8 | rs10090605 | 4818950 | *CSMD1* | C | A | 0.02 | 0 | 2.95E-08 | 10.05 (4.44-22.71) |
| 8 | rs12542818 | 4817813 | *CSMD1* | A | C | 0.02 | 0 | 2.95E-08 | 10.05 (4.44-22.71) |
| 8 | rs12549291 | 4813349 | *CSMD1* | T | G | 0.02 | 0 | 3.18E-08 | 9.99 (4.42-22.58) |
| 7 | rs2402961 | 129405681 | *UBE2H* | C | T | 0.06 | 0 | 5.49E-08 | 3.82 (2.36-6.20) |
| 12 | rs10785655 | 47493483 | *LOC100233209* | A | G | 0.02 | 0 | 1.36E-07 | 8.06 (3.71-17.50) |
| 7 | rs10275592 | 129248502 | *NRF1* | A | G | 0.06 | 0 | 2.63E-07 | 3.52 (2.18-5.67) |
| 3 | rs10510373 | 7911027 | *LMCD1* | C | G | 0.04 | 0 | 5.42E-07 | 4.15 (2.38-7.25) |
| 7 | rs2402972 | 129381953 | *NRF1* | T | C | 0.06 | 0 | 1.04E-06 | 3.37 (2.07-5.50) |
| 7 | rs6958958 | 129358476 | *NRF1* | C | T | 0.06 | 0 | 1.36E-06 | 3.24 (2.01-5.23) |
| 2 | rs7558738 | 38054087 | *FAM82A1* | T | C | 0.32 | 0.14 | 1.64E-06 | 1.82 (1.42-2.32) |
| 13 | rs41360252 | 23922462 | *SACS* | C | T | 0.01 | 0 | 1.66E-06 | 13.29 (4.61-38.30) |
| 20 | rs2185558 | 36905894 | *BPI* | C | T | 0.22 | 0.07 | 1.84E-06 | 1.85 (1.44-2.39) |
| 9 | rs17086413 | 86139397 | *FRMD3* | C | G | 0.22 | 0.09 | 2.47E-06 | 2.03 (1.51-2.72) |
| 10 | rs7074909 | 24841274 | *ARHGAP21* | G | A | 0.09 | 0.04 | 2.64E-06 | 2.69 (1.78-4.06) |
| 6 | rs12660680 | 65292039 | *EYS* | G | A | 0.17 | 0.41 | 3.28E-06 | 0.49 (0.36-0.66) |
| 2 | rs10183499 | 38056472 | *FAM82A1* | C | G | 0.32 | 0.16 | 3.49E-06 | 1.77 (1.39-2.24) |
| 6 | rs582535 | 65309771 | *EYS* | G | A | 0.16 | 0.39 | 4.02E-06 | 0.48 (0.35-0.66) |
| 6 | rs12663622 | 65301787 | *EYS* | G | C | 0.16 | 0.39 | 4.72E-06 | 0.47 (0.34-0.65) |
| 2 | rs4143272 | 38059334 | *FAM82A1* | T | G | 0.31 | 0.16 | 4.85E-06 | 1.75 (1.38-2.22) |
| 6 | rs12660387 | 65291644 | *EYS* | G | A | 0.17 | 0.39 | 4.90E-06 | 0.49 (0.36-0.66) |
| 6 | rs12662610 | 65301824 | *EYS* | C | T | 0.17 | 0.39 | 4.90E-06 | 0.49 (0.36-0.66) |
| 6 | rs17217924 | 65288688 | *EYS* | G | A | 0.17 | 0.39 | 4.90E-06 | 0.49 (0.36-0.66) |
| 6 | rs34148654 | 65295448 | *EYS* | T | C | 0.17 | 0.39 | 4.90E-06 | 0.49 (0.36-0.66) |
| 11 | rs7108263 | 134757882 | *B3GAT1* | A | G | 0.02 | 0 | 4.98E-06 | 7.36 (3.13-17.35) |
| 1 | rs1481397 | 105303590 | *PRMT6* | A | G | 0.19 | 0.06 | 5.19E-06 | 1.88 (1.43-2.46) |
| 1 | rs7523359 | 105324488 | *PRMT6* | T | C | 0.19 | 0.06 | 5.36E-06 | 1.87 (1.43-2.46) |
| 6 | rs1328214 | 65286643 | *EYS* | T | C | 0.28 | 0.54 | 5.47E-06 | 0.56 (0.43-0.72) |
| 19 | rs681059 | 16269507 | *CIB3* | C | T | 0.02 | 0 | 5.53E-06 | 6.24 (2.83-13.74) |
| 12 | rs11063868 | 721388 | *NINJ2* | A | G | 0.05 | 0 | 6.37E-06 | 3.54 (2.05-6.14) |
| 1 | rs1871782 | 105304918 | *PRMT6* | T | G | 0.19 | 0.06 | 6.56E-06 | 1.86 (1.42-2.45) |
| 15 | rs16966249 | 38419470 | *SPRED1* | T | G | 0.12 | 0.01 | 7.20E-06 | 2.10 (1.52-2.90) |
| 15 | rs7176853 | 38421410 | *SPRED1* | T | A | 0.12 | 0.01 | 7.20E-06 | 2.10 (1.52-2.90) |
| 12 | rs1522732 | 72610044 | *LOC283392* | A | C | 0.03 | 0 | 7.62E-06 | 5.01 (2.47-10.13) |
| 7 | rs9792084 | 129244665 | *NRF1* | T | C | 0.08 | 0 | 7.67E-06 | 2.21 (1.56-3.13) |
| 7 | rs7805465 | 129248638 | *NRF1* | A | G | 0.07 | 0 | 8.12E-06 | 2.87 (1.81-4.56) |
| 2 | rs4670222 | 38054623 | *FAM82A1* | T | C | 0.33 | 0.16 | 9.14E-06 | 1.69 (1.34-2.13) |
| 10 | rs10764785 | 130319747 | *MGMT* | A | C | 0.32 | 0.14 | 9.77E-06 | 1.67 (1.33-2.10) |
| 11 | rs1944032 | 76775472 | *CAPN5* | C | T | 0.41 | 0.18 | 1.03E-05 | 1.60 (1.30-1.97) |
| 1 | rs1041311 | 205456028 | *PCTK3* | A | T | 0.33 | 0.11 | 1.04E-05 | 1.71 (1.35-2.17) |
| 6 | rs12661208 | 65302728 | *EYS* | G | C | 0.17 | 0.38 | 1.07E-05 | 0.50 (0.37-0.68) |
| 5 | rs17335879 | 39463197 | *PTGER4* | A | G | 0.12 | 0.03 | 1.11E-05 | 1.94 (1.44-2.61) |
| 1 | rs1823549 | 103147831 | *COL11A1* | A | G | 0.02 | 0 | 1.11E-05 | 6.13 (2.73-13.76) |
| 1 | rs2376175 | 103155901 | *COL11A1* | T | C | 0.02 | 0 | 1.11E-05 | 6.13 (2.73-13.76) |
| 6 | rs10944599 | 65292620 | *EYS* | A | G | 0.17 | 0.39 | 1.12E-05 | 0.50 (0.36-0.68) |
| 1 | rs12043310 | 105332372 | *PRMT6* | T | C | 0.17 | 0.03 | 1.19E-05 | 2.01 (1.47-2.75) |
| 10 | rs10829419 | 130318217 | *MGMT* | C | T | 0.30 | 0.14 | 1.20E-05 | 1.69 (1.34-2.13) |
| 12 | rs16926687 | 23886872 | *SOX5* | A | G | 0.02 | 0 | 1.29E-05 | 7.67 (3.07-19.18) |
| 12 | rs9805112 | 23912046 | *SOX5* | C | T | 0.02 | 0 | 1.29E-05 | 7.67 (3.07-19.18) |
| 6 | rs16881244 | 51148528 | *WTAP* | A | G | 0.05 | 0 | 1.29E-05 | 3.29 (1.93-5.63) |
| 14 | rs17825295 | 94910349 | *SERPINA11* | T | G | 0.02 | 0 | 1.53E-05 | 7.98 (3.11-20.45) |
| 14 | rs17825343 | 94916315 | *SERPINA11* | C | T | 0.02 | 0 | 1.53E-05 | 7.98 (3.11-20.45) |
| 14 | rs17825476 | 94933091 | *SERPINA9* | A | C | 0.02 | 0 | 1.53E-05 | 7.98 (3.11-20.45) |
| 11 | rs308335 | 67776686 | *ALDH3B1* | G | A | 0.02 | 0 | 1.58E-05 | 5.03 (2.42-10.47) |
| 2 | rs12104501 | 53214587 | *ASB3* | A | C | 0.03 | 0 | 1.61E-05 | 3.99 (2.13-7.48) |
| 2 | rs1864432 | 38064671 | *FAM82A1* | T | C | 0.30 | 0.16 | 1.77E-05 | 1.69 (1.33-2.15) |
| 1 | rs4951002 | 205452302 | *PCTK3* | T | G | 0.32 | 0.11 | 1.77E-05 | 1.72 (1.34-2.20) |
| 19 | rs4349264 | 48531332 | *CABP5* | C | G | 0.01 | 0 | 1.92E-05 | 10.01 (3.48-28.8) |
| 7 | rs4731621 | 129374657 | *NRF1* | T | C | 0.06 | 0 | 1.97E-05 | 2.88 (1.77-4.69) |
| 1 | rs2798413 | 166227854 | *FMO9P* | C | T | 0.33 | 0.60 | 2.01E-05 | 0.60 (0.47-0.76) |
| 1 | rs12090819 | 102951488 | *COL11A1* | G | A | 0.02 | 0 | 2.06E-05 | 6.58 (2.76-15.67) |
| 1 | rs12036613 | 105325538 | *PRMT6* | T | C | 0.16 | 0.04 | 2.09E-05 | 1.97 (1.44-2.68) |
| 1 | rs12042214 | 105344196 | *PRMT6* | G | A | 0.16 | 0.04 | 2.09E-05 | 1.97 (1.44-2.68) |
| 1 | rs4290071 | 105344132 | *PRMT6* | T | C | 0.16 | 0.04 | 2.26E-05 | 1.97 (1.44-2.69) |
| 16 | rs1558680 | 51566155 | *HNRPA1L-2* | C | G | 0.14 | 0.05 | 2.31E-05 | 1.91 (1.42-2.58) |
| 2 | rs736019 | 181526568 | *UBE2E3* | C | A | 0.41 | 0.23 | 2.33E-05 | 1.64 (1.30-2.07) |
| 16 | rs11647783 | 83822419 | *CDH13* | G | C | 0.08 | 0.03 | 2.50E-05 | 2.09 (1.48-2.95) |
| 7 | rs7810045 | 88742607 | *ZNF804B* | T | C | 0.02 | 0 | 2.53E-05 | 5.35 (2.45-11.66) |
| 4 | rs7686062 | 154445527 | *KIAA0922* | T | G | 0.14 | 0.04 | 2.60E-05 | 2.08 (1.48-2.92) |
| 1 | rs1193096 | 208195516 | *PLXNA2* | T | G | 0.08 | 0.03 | 2.62E-05 | 2.39 (1.59-3.60) |
| 4 | rs16871181 | 10641579 | *CLNK* | C | T | 0.22 | 0.06 | 2.65E-05 | 1.76 (1.35-2.29) |
| 1 | rs10785715 | 105350772 | *PRMT6* | G | T | 0.16 | 0.04 | 2.69E-05 | 1.96 (1.43-2.67) |
| 1 | rs10918416 | 166221811 | *FMO9P* | C | A | 0.31 | 0.59 | 2.70E-05 | 0.61 (0.48-0.77) |
| 2 | rs2707243 | 38071114 | *FAM82A1* | T | C | 0.35 | 0.18 | 2.74E-05 | 1.64 (1.30-2.07) |
| 9 | rs4504713 | 86137112 | *FRMD3* | C | T | 0.18 | 0.07 | 2.80E-05 | 1.90 (1.41-2.57) |
| 2 | rs17489442 | 38073231 | *FAM82A1* | A | G | 0.36 | 0.19 | 2.88E-05 | 1.64 (1.30-2.06) |
| 2 | rs10202422 | 181524944 | *UBE2E3* | C | T | 0.41 | 0.23 | 3.05E-05 | 1.63 (1.29-2.05) |
| 2 | rs12712559 | 38073477 | *FAM82A1* | C | T | 0.36 | 0.19 | 3.09E-05 | 1.64 (1.30-2.06) |
| 2 | rs11691648 | 190800035 | *MSTN* | A | G | 0.23 | 0.09 | 3.13E-05 | 1.66 (1.31-2.11) |
| 16 | rs3851775 | 29279750 | *RUNDC2C* | G | A | 0.50 | 0.18 | 3.18E-05 | 1.61 (1.28-2.01) |
| 3 | rs9990098 | 45413536 | *LARS2* | A | G | 0.30 | 0.54 | 3.23E-05 | 0.59 (0.46-0.76) |
| 1 | rs7524755 | 165694897 | *TMCO1* | A | G | 0.01 | 0 | 3.31E-05 | 9.29 (3.24-26.63) |
| 1 | rs4950485 | 147240042 | *GJA5* | G | A | 0.33 | 0.56 | 3.59E-05 | 0.61 (0.49-0.77) |
| 11 | rs10793234 | 76780975 | *CAPN5* | A | G | 0.50 | 0.27 | 3.60E-05 | 1.54 (1.26-1.89) |
| 15 | rs9944168 | 57862121 | *GCOM1* | G | A | 0.46 | 0.62 | 3.67E-05 | 0.63 (0.50-0.78) |
| 10 | rs16924889 | 24843786 | *ARHGAP21* | T | C | 0.09 | 0.04 | 3.87E-05 | 2.36 (1.57-3.56) |
| 1 | rs7528732 | 103200189 | *COL11A1* | G | A | 0.02 | 0 | 3.98E-05 | 6.1 (2.57-14.45) |
| 11 | rs10768245 | 37102463 | *LRRC4C* | T | G | 0.32 | 0.56 | 4.18E-05 | 0.58 (0.45-0.75) |
| 15 | rs4779031 | 79533372 | *ANKRD34C* | C | A | 0.18 | 0.09 | 4.35E-05 | 1.89 (1.39-2.57) |
| 1 | rs1317353 | 22706579 | *ZBTB40* | T | C | 0.26 | 0.07 | 4.52E-05 | 1.63 (1.29-2.06) |
| 11 | rs680730 | 117475233 | *DSCAML1* | T | C | 0.36 | 0.23 | 4.53E-05 | 1.70 (1.32-2.19) |
| 8 | rs12165 | 26269412 | *BNIP3L* | C | T | 0.15 | 0.04 | 4.62E-05 | 1.99 (1.43-2.76) |
| 1 | rs17111440 | 85021277 | *CTBS* | G | T | 0.08 | 0.19 | 4.64E-05 | 0.42 (0.28-0.64) |
| 5 | rs13171149 | 9182717 | *SEMA5A* | C | T | 0.07 | 0 | 4.65E-05 | 2.62 (1.65-4.16) |
| 5 | rs17653473 | 149183376 | *PPARGC1B* | T | G | 0.29 | 0.20 | 4.69E-05 | 1.66 (1.30-2.13) |
| 1 | rs9435825 | 229359336 | *RAB4A* | C | A | 0.05 | 0 | 4.71E-05 | 3.11 (1.80-5.38) |
| 15 | rs9944289 | 57862014 | *GCOM1* | C | T | 0.46 | 0.61 | 4.78E-05 | 0.63 (0.51-0.79) |
| 1 | rs17111498 | 85028274 | *CTBS* | C | A | 0.08 | 0.19 | 4.85E-05 | 0.42 (0.28-0.64) |
| 10 | rs11014149 | 24838797 | *ARHGAP21* | G | A | 0.08 | 0.04 | 5.11E-05 | 2.42 (1.58-3.70) |
| 3 | rs10049401 | 168413945 | *MECOM* | C | T | 0.02 | 0 | 5.14E-05 | 4.01 (2.05-7.85) |
| 3 | rs475749 | 168456385 | *MECOM* | G | A | 0.02 | 0 | 5.14E-05 | 4.01 (2.05-7.85) |
| 3 | rs535028 | 168496750 | *MECOM* | G | A | 0.02 | 0 | 5.14E-05 | 4.01 (2.05-7.85) |
| 1 | rs7540036 | 205452224 | *PCTK3* | C | G | 0.32 | 0.13 | 5.16E-05 | 1.65 (1.30-2.11) |
| 1 | rs1528858 | 214291028 | *SMYD2* | T | C | 0.04 | 0 | 5.18E-05 | 3.16 (1.81-5.51) |
| 1 | rs12138359 | 115681071 | *NGF* | A | G | 0.14 | 0.09 | 5.21E-05 | 1.93 (1.40-2.64) |
| 2 | rs6431421 | 236949119 | *AGAP1* | C | T | 0.12 | 0.03 | 5.45E-05 | 2.03 (1.44-2.85) |
| 13 | rs17072706 | 49674929 | *FNDC3A* | T | A | 0.11 | 0.04 | 5.55E-05 | 2.01 (1.43-2.82) |
| 11 | rs7122948 | 74800829 | *SLCO2B1* | C | T | 0.44 | 0.19 | 6.02E-05 | 1.62 (1.28-2.06) |
| 16 | rs935754 | 60377491 | *CDH8* | G | A | 0.03 | 0 | 6.08E-05 | 3.91 (2.01-7.61) |
| 5 | rs12652630 | 67443534 | *PIK3R1* | G | A | 0.36 | 0.49 | 6.14E-05 | 0.61 (0.47-0.77) |
| 1 | rs7532083 | 235042641 | *TOMM20* | T | C | 0.22 | 0.34 | 6.15E-05 | 0.55 (0.41-0.74) |
| 7 | rs13223537 | 94966167 | *PON3* | C | T | 0.19 | 0.04 | 6.15E-05 | 1.93 (1.40-2.65) |
| 17 | rs17510939 | 11169773 | *SHISA6* | T | C | 0.08 | 0.03 | 6.21E-05 | 2.26 (1.52-3.37) |
| 11 | rs10899068 | 74785720 | *NPM1* | A | G | 0.44 | 0.19 | 6.35E-05 | 1.62 (1.28-2.06) |
| 2 | rs2123508 | 236944860 | *AGAP1* | T | C | 0.12 | 0.03 | 6.36E-05 | 2.02 (1.43-2.85) |
| 2 | rs2123509 | 236944801 | *AGAP1* | A | G | 0.12 | 0.03 | 6.36E-05 | 2.02 (1.43-2.85) |
| 1 | rs12134493 | 115677946 | *NGF* | T | G | 0.14 | 0.09 | 6.45E-05 | 1.91 (1.39-2.62) |
| 1 | rs10918393 | 166184824 | *FMO9P* | G | A | 0.32 | 0.56 | 6.50E-05 | 0.61 (0.48-0.78) |
| 9 | rs4242621 | 85955595 | *FRMD3* | C | T | 0.26 | 0.13 | 6.52E-05 | 1.73 (1.32-2.26) |
| 4 | rs17467937 | 10617438 | *CLNK* | A | T | 0.21 | 0.06 | 6.80E-05 | 1.72 (1.32-2.24) |
| 1 | rs11811929 | 105396240 | *PRMT6* | A | G | 0.17 | 0.04 | 7.01E-05 | 1.80 (1.35-2.40) |
| 12 | rs4768773 | 47498533 | *LOC100233209* | G | A | 0.04 | 0 | 7.13E-05 | 3.36 (1.85-6.10) |
| 3 | rs17182582 | 132072419 | *ACPP* | C | G | 0.02 | 0 | 7.16E-05 | 5.41 (2.35-12.44) |
| 10 | rs10752350 | 15104215 | *OLAH* | T | C | 0.30 | 0.56 | 7.16E-05 | 0.62 (0.49-0.79) |
| 5 | rs17099388 | 142095250 | *ARHGAP26* | C | T | 0.52 | 0.37 | 7.38E-05 | 1.61 (1.27-2.04) |
| 5 | rs13187858 | 39476930 | *PTGER4* | C | A | 0.12 | 0.03 | 7.39E-05 | 1.82 (1.35-2.45) |
| 10 | rs17095891 | 118973556 | *SLC18A2* | T | C | 0.15 | 0.03 | 7.40E-05 | 1.84 (1.36-2.49) |
| 10 | rs363313 | 118967460 | *KCNK18* | A | G | 0.15 | 0.03 | 7.40E-05 | 1.84 (1.36-2.49) |
| 10 | rs3858335 | 118973145 | *SLC18A2* | A | C | 0.15 | 0.03 | 7.40E-05 | 1.84 (1.36-2.49) |
| 11 | rs7950506 | 76752248 | *B3GNT6* | T | C | 0.38 | 0.18 | 7.58E-05 | 1.53 (1.24-1.89) |
| 16 | rs17707862 | 83829805 | *CDH13* | A | G | 0.08 | 0.03 | 7.69E-05 | 2.28 (1.52-3.43) |
| 1 | rs10918403 | 166206473 | *FMO9P* | C | T | 0.31 | 0.57 | 7.72E-05 | 0.63 (0.50-0.79) |
| 10 | rs11259457 | 15107077 | *OLAH* | T | C | 0.28 | 0.54 | 7.72E-05 | 0.62 (0.49-0.79) |
| 1 | rs12126584 | 84956858 | *BXDC5* | C | T | 0.09 | 0.17 | 7.76E-05 | 0.43 (0.29-0.66) |
| 1 | rs3813605 | 84970947 | *GNG5* | T | C | 0.09 | 0.17 | 7.76E-05 | 0.43 (0.29-0.66) |
| 1 | rs7514763 | 84930166 | *BXDC5* | G | T | 0.09 | 0.17 | 7.76E-05 | 0.43 (0.29-0.66) |
| 3 | rs13314784 | 45406592 | *LARS2* | G | A | 0.13 | 0.06 | 7.79E-05 | 1.94 (1.40-2.70) |
| 12 | rs10861285 | 105263238 | *SLC41A2* | T | G | 0.08 | 0.03 | 8.03E-05 | 2.28 (1.51-3.44) |
| 12 | rs11112216 | 105262947 | *SLC41A2* | T | C | 0.08 | 0.03 | 8.03E-05 | 2.28 (1.51-3.44) |
| 3 | rs17637338 | 45413607 | *LARS2* | T | C | 0.30 | 0.53 | 8.06E-05 | 0.60 (0.47-0.77) |
| 1 | rs12031590 | 182722554 | *NPL* | G | A | 0.38 | 0.53 | 8.45E-05 | 0.58 (0.44-0.76) |
| 1 | rs17110519 | 84968749 | *GNG5* | T | G | 0.09 | 0.17 | 8.54E-05 | 0.44 (0.29-0.66) |
| 1 | rs2802986 | 235050025 | *TOMM20* | C | T | 0.49 | 0.33 | 8.62E-05 | 1.61 (1.27-2.04) |
| 17 | rs11653801 | 54834041 | *C17orf67* | C | G | 0.02 | 0 | 8.64E-05 | 6.31 (2.51-15.81) |
| 11 | rs6589285 | 112264620 | *NCAM1* | G | A | 0.45 | 0.31 | 8.81E-05 | 1.54 (1.24-1.91) |
| 1 | rs12038712 | 105395417 | *PRMT6* | G | A | 0.17 | 0.04 | 8.86E-05 | 1.78 (1.33-2.38) |
| 3 | rs2034007 | 19371858 | *KCNH8* | T | C | 0.47 | 0.22 | 8.88E-05 | 1.58 (1.26-1.99) |
| 8 | rs3808580 | 26250296 | *BNIP3L* | T | C | 0.14 | 0.04 | 8.89E-05 | 1.94 (1.39-2.71) |
| 4 | rs6845149 | 10634161 | *CLNK* | C | A | 0.22 | 0.06 | 8.90E-05 | 1.70 (1.30-2.22) |
| 10 | rs17095821 | 118959363 | *KCNK18* | T | C | 0.14 | 0.03 | 9.28E-05 | 1.85 (1.36-2.52) |
| 10 | rs3847483 | 118959013 | *KCNK18* | T | C | 0.14 | 0.03 | 9.28E-05 | 1.85 (1.36-2.52) |
| 9 | rs16916521 | 114743339 | *SUSD1* | T | C | 0.28 | 0.09 | 9.29E-05 | 1.64 (1.28-2.09) |
| 1 | rs6703824 | 147241002 | *GJA5* | T | C | 0.33 | 0.54 | 9.38E-05 | 0.63 (0.50-0.80) |
| 7 | rs10274562 | 11222521 | *THSD7A* | A | G | 0.26 | 0.11 | 9.44E-05 | 1.61 (1.27-2.05) |
| 3 | rs9840170 | 133827144 | *RYK* | G | A | 0.19 | 0.34 | 9.58E-05 | 0.56 (0.42-0.75) |
| 20 | rs6057602 | 31181808 | *LOC284805* | A | C | 0.10 | 0.04 | 9.67E-05 | 2.08 (1.44-3.02) |
| 10 | rs4881156 | 3368596 | *KLF6* | A | G | 0.32 | 0.46 | 9.71E-05 | 0.55 (0.40-0.74) |
| 3 | rs9846871 | 133852998 | *RYK* | G | T | 0.20 | 0.34 | 9.82E-05 | 0.56 (0.42-0.75) |
| 5 | rs12521269 | 119260377 | *PRR16* | T | A | 0.01 | 0 | 9.85E-05 | 7.75 (2.76-21.7) |
| 8 | rs2046223 | 26210178 | *PPP2R2A* | T | C | 0.14 | 0.06 | 9.98E-05 | 1.93 (1.39-2.70) |
| 14 | rs12435173 | 97094944 | *VRK1* | G | A | 0.33 | 0.14 | 1.00E-04 | 1.66 (1.28-2.13) |

MAF, minor allele frequency; HR, hazard ratio; CI, confidence interval; SNP, single nucleotide polymorphism.

* Physical position based on human reference genome build hg 19 (GRCh37).

**Table S4. List of SNPs (n=135) associated with major molecular response achievement following imatinib therapy in the discovery set (*p* < 1.0E-04)**

| **Chromosome** | **SNP** | **Position*** | **Nearby Gene** | **Minor**  **allele** | **Major**  **allele** | **MAF**  **in Response** | **MAF**  **in Non-response** | ***p* value** | **HR (CI, 95%)** |
| --- | --- | --- | --- | --- | --- | --- | --- | --- | --- |
| 6 | rs1556879 | 150615876 | *IYD* | A | G | 0.02 | 0 | 4.81E-08 | 17.10 (6.17-47.41) |
| 12 | rs7972648 | 26790894 | *ITPR2* | G | T | 0.07 | 0.01 | 5.54E-08 | 3.21 (2.11-4.88) |
| 10 | rs6583938 | 96083515 | *PLCE1* | T | A | 0.02 | 0 | 1.69E-07 | 13.06 (4.99-34.22) |
| 10 | rs7074909 | 24841274 | *ARHGAP21* | G | A | 0.10 | 0.03 | 1.75E-07 | 3.08 (2.02-4.69) |
| 2 | rs17040511 | 50605863 | *NRXN1* | G | C | 0.05 | 0 | 1.95E-07 | 4.67 (2.61-8.34) |
| 12 | rs1463590 | 26793755 | *ITPR2* | G | A | 0.05 | 0.01 | 3.93E-07 | 4.36 (2.47-7.71) |
| 16 | rs8064066 | 78539115 | *WWOX* | C | G | 0.16 | 0.04 | 1.04E-06 | 2.10 (1.56-2.82) |
| 1 | rs1528858 | 214291028 | *SMYD2* | T | C | 0.05 | 0.01 | 1.05E-06 | 4.32 (2.40-7.76) |
| 16 | rs2667599 | 78539722 | *WWOX* | G | C | 0.15 | 0.04 | 1.58E-06 | 2.15 (1.57-2.94) |
| 16 | rs2738740 | 78544042 | *WWOX* | G | T | 0.15 | 0.04 | 2.26E-06 | 2.16 (1.57-2.97) |
| 1 | rs12745898 | 210815017 | *HHAT* | A | G | 0.01 | 0.01 | 2.40E-06 | 20.61 (5.86-72.49) |
| 16 | rs1397927 | 78538355 | *WWOX* | A | G | 0.15 | 0.04 | 2.87E-06 | 2.14 (1.56-2.95) |
| 17 | rs9896009 | 3021996 | *OR1G1* | T | C | 0.01 | 0.01 | 3.27E-06 | 21.44 (5.89-77.99) |
| 11 | rs7125210 | 67579039 | *LOC389634* | T | G | 0.01 | 0 | 4.03E-06 | 12.42 (4.26-36.26) |
| 16 | rs2738742 | 78548656 | *WWOX* | A | T | 0.14 | 0.04 | 4.38E-06 | 2.13 (1.54-2.93) |
| 7 | rs17157274 | 109585662 | *EIF3IP1* | C | A | 0.25 | 0.13 | 5.30E-06 | 1.86 (1.42-2.43) |
| 16 | rs11150083 | 78546338 | *WWOX* | C | G | 0.14 | 0.04 | 5.38E-06 | 2.11 (1.53-2.91) |
| 16 | rs2667608 | 78548310 | *WWOX* | C | T | 0.14 | 0.04 | 5.38E-06 | 2.11 (1.53-2.91) |
| 16 | rs2667609 | 78548574 | *WWOX* | C | A | 0.14 | 0.04 | 5.38E-06 | 2.11 (1.53-2.91) |
| 4 | rs17044483 | 165029168 | *LOC723972* | A | T | 0.07 | 0.03 | 6.28E-06 | 3.09 (1.90-5.05) |
| 10 | rs16924889 | 24843786 | *ARHGAP21* | T | C | 0.10 | 0.03 | 6.55E-06 | 2.63 (1.73-4.00) |
| 18 | rs641205 | 4242200 | *LOC642597* | T | C | 0.11 | 0.01 | 6.90E-06 | 2.26 (1.58-3.21) |
| 12 | rs10847412 | 128048447 | *TMEM132C* | C | A | 0.05 | 0.02 | 7.19E-06 | 3.82 (2.13-6.85) |
| 12 | rs11059265 | 128052533 | *TMEM132C* | G | C | 0.05 | 0.02 | 7.19E-06 | 3.82 (2.13-6.85) |
| 8 | rs7018080 | 1301757 | *DLGAP2* | A | C | 0.20 | 0.11 | 8.41E-06 | 2.11 (1.52-2.92) |
| 8 | rs1849306 | 23436323 | *NKX3-1* | A | G | 0.04 | 0 | 8.53E-06 | 4.26 (2.25-8.06) |
| 8 | rs17152930 | 11010826 | *XKR6* | T | C | 0.01 | 0 | 9.15E-06 | 10.64 (3.74-30.22) |
| 18 | rs2032160 | 4236531 | *LOC642597* | G | T | 0.11 | 0.01 | 9.40E-06 | 2.24 (1.57-3.20) |
| 3 | rs2707985 | 142599820 | *PCOLCE2* | G | A | 0.23 | 0.10 | 1.05E-05 | 1.82 (1.40-2.38) |
| 18 | rs1473631 | 4236056 | *LOC642597* | T | G | 0.12 | 0.01 | 1.13E-05 | 2.20 (1.55-3.12) |
| 9 | rs11139399 | 84372741 | *FLJ44082* | G | A | 0.42 | 0.27 | 1.40E-05 | 1.75 (1.36-2.26) |
| 6 | rs6919280 | 103684742 | *HACE1* | T | C | 0.03 | 0 | 1.48E-05 | 4.85 (2.37-9.90) |
| 15 | rs17603016 | 86818771 | *AGBL1* | T | C | 0.04 | 0 | 1.49E-05 | 3.58 (2.01-6.37) |
| 4 | rs4264788 | 164987994 | *LOC723972* | A | G | 0.08 | 0.03 | 1.50E-05 | 2.88 (1.78-4.65) |
| 18 | rs16946303 | 4240886 | *LOC642597* | C | T | 0.12 | 0.01 | 1.55E-05 | 2.18 (1.53-3.10) |
| 3 | rs9846063 | 81880937 | *CYP51A1* | T | G | 0.09 | 0.02 | 1.76E-05 | 2.62 (1.69-4.06) |
| 1 | rs12562339 | 82529612 | *TTLL7* | T | C | 0.16 | 0.04 | 1.76E-05 | 2.09 (1.49-2.93) |
| 2 | rs6732799 | 106401954 | *NCK2* | T | C | 0.23 | 0.11 | 1.80E-05 | 1.83 (1.39-2.42) |
| 10 | rs11014149 | 24838797 | *ARHGAP21* | G | A | 0.09 | 0.03 | 1.86E-05 | 2.59 (1.68-4.01) |
| 10 | rs17498553 | 122501681 | *BRWD2* | A | T | 0.05 | 0 | 1.89E-05 | 3.28 (1.90-5.66) |
| 10 | rs11201618 | 87186017 | *GRID1* | A | G | 0.02 | 0 | 1.93E-05 | 7.67 (3.01-19.52) |
| 4 | rs17013921 | 129835710 | *SCLT1* | A | G | 0.01 | 0.01 | 1.94E-05 | 15.13 (4.35-52.60) |
| 10 | rs11201609 | 87167533 | *GRID1* | T | C | 0.02 | 0 | 2.15E-05 | 7.55 (2.97-19.20) |
| 8 | rs1866347 | 23547510 | *NKX2-6* | A | G | 0.02 | 0 | 2.15E-05 | 7.55 (2.97-19.20) |
| 10 | rs6560751 | 1683641 | *ADARB2* | T | C | 0.02 | 0 | 2.24E-05 | 6.24 (2.68-14.54) |
| 18 | rs584328 | 8899596 | *NDUFV2* | T | G | 0.32 | 0.14 | 2.27E-05 | 1.78 (1.37-2.33) |
| 16 | rs17720649 | 78552528 | *WWOX* | A | G | 0.14 | 0.04 | 2.35E-05 | 2.03 (1.46-2.81) |
| 10 | rs4752530 | 123081449 | *FGFR2* | A | C | 0.25 | 0.39 | 2.35E-05 | 0.55 (0.41-0.72) |
| 22 | rs4823197 | 44557063 | *PARVB* | T | C | 0.01 | 0 | 2.38E-05 | 9.61 (3.37-27.46) |
| 16 | rs2738710 | 78524099 | *WWOX* | C | T | 0.25 | 0.13 | 2.41E-05 | 1.87 (1.40-2.50) |
| 1 | rs614020 | 57419287 | *C8B* | C | T | 0.42 | 0.61 | 2.87E-05 | 0.59 (0.46-0.76) |
| 10 | rs11201614 | 87173351 | *GRID1* | A | G | 0.01 | 0 | 2.94E-05 | 9.10 (3.23-25.65) |
| 17 | rs12603425 | 53576069 | *TMEM100* | C | T | 0.26 | 0.16 | 3.05E-05 | 1.79 (1.36-2.35) |
| 3 | rs9821424 | 145734131 | *PLOD2* | T | A | 0.21 | 0.07 | 3.06E-05 | 1.81 (1.37-2.39) |
| 3 | rs2581637 | 142604027 | *PCOLCE2* | C | G | 0.23 | 0.11 | 3.13E-05 | 1.77 (1.35-2.32) |
| 3 | rs1318819 | 10438649 | *ATP2B2* | A | G | 0.25 | 0.22 | 3.23E-05 | 1.97 (1.43-2.71) |
| 16 | rs16952652 | 80248426 | *DYNLRB2* | G | C | 0.01 | 0 | 3.50E-05 | 9.08 (3.19-25.81) |
| 16 | rs1877282 | 78539515 | *WWOX* | C | G | 0.22 | 0.08 | 3.59E-05 | 1.83 (1.37-2.44) |
| 10 | rs7096842 | 64583441 | *NRBF2* | T | C | 0.03 | 0 | 3.59E-05 | 5.33 (2.41-11.78) |
| 8 | rs10104250 | 23431192 | *NKX3-1* | A | C | 0.03 | 0 | 3.61E-05 | 4.68 (2.25-9.73) |
| 1 | rs11163417 | 82517645 | *TTLL7* | T | C | 0.22 | 0.08 | 3.70E-05 | 1.87 (1.39-2.52) |
| 9 | rs7034563 | 81809181 | *CHCHD2* | G | C | 0.27 | 0.11 | 3.86E-05 | 1.79 (1.35-2.35) |
| 4 | rs1706214 | 34873377 | *ARAP2* | G | T | 0.05 | 0.02 | 3.87E-05 | 3.18 (1.83-5.51) |
| 16 | rs11643772 | 20402499 | *PDILT* | G | T | 0.10 | 0.03 | 3.97E-05 | 2.21 (1.52-3.23) |
| 6 | rs527168 | 10550149 | *GCNT2* | C | T | 0.13 | 0.31 | 3.98E-05 | 0.47 (0.33-0.67) |
| 8 | rs4872085 | 23075488 | *TNFRSF10A* | C | G | 0.29 | 0.43 | 4.01E-05 | 0.56 (0.43-0.74) |
| 18 | rs4399633 | 67331750 | *DOK6* | G | T | 0.02 | 0 | 4.10E-05 | 7.11 (2.78-18.14) |
| 16 | rs254315 | 83756412 | *CDH13* | G | A | 0.15 | 0.08 | 4.30E-05 | 2.05 (1.45-2.90) |
| 1 | rs2501839 | 212826272 | *BATF3* | T | G | 0.04 | 0.01 | 4.34E-05 | 4.07 (2.08-7.97) |
| 12 | rs6487565 | 26759254 | *ITPR2* | A | G | 0.04 | 0.02 | 4.38E-05 | 4.05 (2.07-7.91) |
| 16 | rs451871 | 85736429 | *C16orf74* | A | G | 0.23 | 0.40 | 4.40E-05 | 0.54 (0.40-0.73) |
| 10 | rs11199890 | 123075510 | *FGFR2* | A | C | 0.24 | 0.38 | 4.46E-05 | 0.56 (0.42-0.74) |
| 20 | rs6089140 | 30578467 | *XKR7* | C | T | 0.08 | 0.04 | 4.48E-05 | 2.62 (1.65-4.16) |
| 15 | rs2715574 | 26493140 | *GABRB3* | G | A | 0.02 | 0.01 | 4.55E-05 | 6.91 (2.73-17.49) |
| 16 | rs374476 | 83757149 | *CDH13* | C | T | 0.14 | 0.07 | 4.66E-05 | 2.07 (1.46-2.93) |
| 16 | rs10871351 | 78522906 | *WWOX* | C | T | 0.22 | 0.11 | 4.78E-05 | 1.83 (1.37-2.45) |
| 4 | rs1706220 | 34881065 | *ARAP2* | G | C | 0.05 | 0.02 | 4.79E-05 | 3.12 (1.80-5.41) |
| 15 | rs2573697 | 26488219 | *GABRB3* | G | A | 0.02 | 0.01 | 4.80E-05 | 6.87 (2.71-17.38) |
| 1 | rs2456825 | 212829059 | *BATF3* | G | C | 0.03 | 0 | 4.87E-05 | 4.27 (2.12-8.60) |
| 8 | rs6999337 | 70754675 | *PRDM14* | T | A | 0.50 | 0.31 | 4.88E-05 | 1.75 (1.34-2.29) |
| 16 | rs17720666 | 78553933 | *WWOX* | T | G | 0.14 | 0.05 | 4.90E-05 | 1.98 (1.42-2.76) |
| 10 | rs17498576 | 122501727 | *BRWD2* | C | T | 0.05 | 0 | 4.94E-05 | 3.19 (1.82-5.60) |
| 10 | rs2505910 | 29917425 | *SVIL* | T | G | 0.04 | 0.01 | 5.16E-05 | 2.94 (1.75-4.96) |
| 1 | rs649069 | 57419226 | *C8B* | G | T | 0.34 | 0.51 | 5.25E-05 | 0.60 (0.47-0.77) |
| 3 | rs34909 | 10453218 | *ATP2B2* | C | T | 0.29 | 0.24 | 5.47E-05 | 1.80 (1.35-2.40) |
| 2 | rs7576796 | 236770736 | *AGAP1* | G | A | 0.03 | 0 | 5.90E-05 | 4.18 (2.08-8.40) |
| 4 | rs7659353 | 122878371 | *RNF185* | A | G | 0.03 | 0 | 6.10E-05 | 4.15 (2.07-8.31) |
| 1 | rs10518661 | 82381380 | *LPHN2* | G | A | 0.14 | 0.03 | 6.16E-05 | 2.01 (1.43-2.82) |
| 20 | rs467267 | 52678410 | *BCAS1* | T | C | 0.48 | 0.32 | 6.21E-05 | 1.64 (1.29-2.08) |
| 2 | rs13385327 | 124278997 | *CNTNAP5* | T | C | 0.19 | 0.09 | 6.53E-05 | 1.85 (1.37-2.51) |
| 2 | rs17010180 | 124275243 | *CNTNAP5* | T | A | 0.19 | 0.09 | 6.53E-05 | 1.85 (1.37-2.51) |
| 2 | rs17010186 | 124275352 | *CNTNAP5* | C | T | 0.19 | 0.09 | 6.53E-05 | 1.85 (1.37-2.51) |
| 3 | rs13076345 | 158612883 | *IQCJ* | A | G | 0.21 | 0.10 | 6.55E-05 | 1.76 (1.33-2.33) |
| 6 | rs1226003 | 10548517 | *GCNT2* | T | G | 0.14 | 0.32 | 6.62E-05 | 0.50 (0.36-0.70) |
| 12 | rs776424 | 70023791 | *BEST3* | T | G | 0.01 | 0 | 6.66E-05 | 8.35 (2.94-23.71) |
| 9 | rs10969596 | 3033744 | *RFX3* | T | C | 0.03 | 0.01 | 6.68E-05 | 4.98 (2.26-10.97) |
| 18 | rs3114279 | 57363451 | *CCBE1* | T | C | 0.36 | 0.24 | 6.69E-05 | 1.65 (1.29-2.11) |
| 1 | rs11580058 | 213513059 | *PROX1* | A | G | 0.02 | 0 | 6.86E-05 | 5.48 (2.37-12.66) |
| 13 | rs9535608 | 51754949 | *SERPINE3* | T | C | 0.02 | 0 | 7.00E-05 | 5.52 (2.38-12.82) |
| 13 | rs9535611 | 51755319 | *SERPINE3* | A | G | 0.02 | 0 | 7.00E-05 | 5.52 (2.38-12.82) |
| 11 | rs467139 | 72327525 | *PDE2A* | G | A | 0.09 | 0.03 | 7.01E-05 | 2.41 (1.56-3.72) |
| 16 | rs2966031 | 64700368 | *CDH11* | G | A | 0.44 | 0.57 | 7.14E-05 | 0.59 (0.45-0.76) |
| 8 | rs10092233 | 23434732 | *NKX3-1* | G | C | 0.03 | 0 | 7.20E-05 | 4.07 (2.03-8.13) |
| 8 | rs11781415 | 23434151 | *NKX3-1* | A | G | 0.03 | 0 | 7.20E-05 | 4.07 (2.03-8.13) |
| 8 | rs41460846 | 23435916 | *NKX3-1* | T | A | 0.03 | 0 | 7.20E-05 | 4.07 (2.03-8.13) |
| 5 | rs16898804 | 10240409 | *FAM173B* | T | C | 0.01 | 0 | 7.36E-05 | 8.19 (2.89-23.15) |
| 10 | rs11199541 | 122531747 | *BRWD2* | T | C | 0.05 | 0.01 | 7.56E-05 | 3.11 (1.77-5.46) |
| 6 | rs1699015 | 38572590 | *BTBD9* | G | T | 0.04 | 0 | 7.59E-05 | 3.60 (1.91-6.80) |
| 6 | rs1699018 | 38568013 | *BTBD9* | A | G | 0.04 | 0 | 7.59E-05 | 3.60 (1.91-6.80) |
| 18 | rs595107 | 8892906 | *NDUFV2* | A | G | 0.31 | 0.14 | 7.70E-05 | 1.70 (1.31-2.22) |
| 16 | rs10514438 | 78583257 | *WWOX* | A | G | 0.14 | 0.05 | 7.86E-05 | 1.95 (1.40-2.71) |
| 16 | rs2738501 | 78569070 | *WWOX* | A | G | 0.14 | 0.05 | 7.86E-05 | 1.95 (1.40-2.71) |
| 5 | rs11746690 | 149143963 | *PPARGC1B* | A | G | 0.06 | 0.03 | 8.02E-05 | 2.76 (1.67-4.57) |
| 11 | rs10899068 | 74785720 | *NPM1* | A | G | 0.44 | 0.28 | 8.05E-05 | 1.71 (1.31-2.24) |
| 16 | rs375142 | 83747327 | *CDH13* | C | G | 0.14 | 0.08 | 8.17E-05 | 2.01 (1.42-2.85) |
| 12 | rs6486740 | 129442675 | *GLT1D1* | C | T | 0.31 | 0.47 | 8.52E-05 | 0.56 (0.42-0.75) |
| 4 | rs11723135 | 918224 | *GAK* | T | A | 0.42 | 0.25 | 8.57E-05 | 1.62 (1.27-2.06) |
| 6 | rs12528088 | 55355591 | *HMGCLL1* | A | T | 0.49 | 0.43 | 8.59E-05 | 1.61 (1.27-2.04) |
| 6 | rs6459081 | 55352586 | *HMGCLL1* | A | T | 0.49 | 0.43 | 8.59E-05 | 1.61 (1.27-2.04) |
| 6 | rs9475319 | 55361878 | *HMGCLL1* | G | A | 0.49 | 0.43 | 8.59E-05 | 1.61 (1.27-2.04) |
| 6 | rs504083 | 10541213 | *GCNT2* | T | C | 0.13 | 0.30 | 8.61E-05 | 0.50 (0.35-0.71) |
| 1 | rs17106881 | 82120303 | *ST13* | G | C | 0.12 | 0.05 | 8.74E-05 | 2.14 (1.46-3.14) |
| 10 | rs12722489 | 6102012 | *IL2RA* | T | C | 0.15 | 0.05 | 8.74E-05 | 2.05 (1.43-2.94) |
| 3 | rs17366568 | 186570453 | *ADIPOQ* | T | C | 0.03 | 0 | 8.83E-05 | 4.82 (2.2-10.58) |
| 11 | rs7122948 | 74800829 | *SLCO2B1* | C | T | 0.44 | 0.28 | 9.10E-05 | 1.71 (1.31-2.24) |
| 12 | rs4378448 | 126668541 | *TMEM132C* | G | A | 0.04 | 0.01 | 9.16E-05 | 3.48 (1.86-6.49) |
| 17 | rs12450808 | 53576656 | *TMEM100* | T | C | 0.25 | 0.16 | 9.17E-05 | 1.78 (1.33-2.37) |
| 10 | rs11591481 | 130325579 | *MGMT* | G | A | 0.31 | 0.51 | 9.35E-05 | 0.61 (0.47-0.78) |
| 1 | rs6588471 | 53644967 | *CPT2* | A | G | 0.15 | 0.30 | 9.48E-05 | 0.52 (0.37-0.72) |
| 6 | rs4254983 | 38517566 | *BTBD9* | C | T | 0.04 | 0 | 9.62E-05 | 3.72 (1.92-7.21) |
| 16 | rs4133363 | 59098160 | *CDH8* | A | G | 0.04 | 0 | 9.77E-05 | 3.69 (1.91-7.13) |
| 12 | rs11169130 | 50124251 | *TMBIM6* | G | C | 0.06 | 0.05 | 9.77E-05 | 2.66 (1.63-4.35) |
| 6 | rs17570942 | 161936998 | *PARK2* | G | A | 0.03 | 0 | 9.87E-05 | 4.73 (2.16-10.34) |
| 6 | rs17649761 | 161937107 | *PARK2* | C | T | 0.03 | 0 | 9.87E-05 | 4.73 (2.16-10.34) |
| 11 | rs2122803 | 57811884 | *OR9I1* | C | T | 0.10 | 0.03 | 9.96E-05 | 2.09 (1.44-3.03) |

MAF, minor allele frequency; HR, hazard ratio; CI, confidence interval; SNP, single nucleotide polymorphism.

* Physical position based on human reference genome build hg 19 (GRCh37).

**Table S5. No association of eight SNPs with chronic myeloid leukemia susceptibility which has been validated as a marker for deep molecular response following imatinib therapy.**

| **Chromosome** | **SNP** | **Position^*^** | **Minor allele** | **Major allele** | **MAF in cases**  **(N=201)** | **MAF in controls^§^**  **(N=497)** | ***p* value^†^** |
| --- | --- | --- | --- | --- | --- | --- | --- |
| 6 | rs10948926 | 55366347 | T | G | 0.42 | 0.39 | 0.36 |
| 6 | rs10948927 | 55366392 | C | T | 0.42 | 0.39 | 0.36 |
| 6 | rs9370435 | 55369916 | G | C | 0.41 | 0.39 | 0.35 |
| 6 | rs4546489 | 55370236 | A | T | 0.41 | 0.39 | 0.45 |
| 6 | rs4275061 | 55371577 | A | G | 0.42 | 0.39 | 0.36 |
| 6 | rs9475323 | 55371939 | T | C | 0.42 | 0.39 | 0.40 |
| 6 | rs9475327 | 55372270 | A | G | 0.41 | 0.39 | 0.43 |
| 6 | rs9296791 | 55376167 | C | T | 0.42 | 0.39 | 0.36 |

MAF, minor allele frequency; SNP, single nucleotide polymorphism.

^*^ Physical position based on human reference genome build hg 19 (GRCh37).

**^§^** The data has been referred from our previously study, kim *et. al..* (Blood, 2011)

^†^ *p* value was calculated using Cochran-Armitage trend test.

**Table S6. The follow-up duration following imatinib therapy of selected patients in fine-mapping study**

| ID | Genotype^§^ | Achievement of DMR^†^ | Time to DMR^‡^ | Time to last follow-up^¶^ |
| --- | --- | --- | --- | --- |
| KOR_01 | GG | Response | 203 | 1161 |
| KOR_02 | GG | Response | 356 | 1450 |
| KOR_03 | GG | Response | 76 | 2504 |
| KOR_04 | GG | Response | 341 | 1166 |
| KOR_05 | GG | Response | 444 | 2366 |
| KOR_06 | GG | Response | 283 | 1549 |
| KOR_07 | GG | Response | 166 | 1446 |
| KOR_08 | GG | Response | 505 | 505 |
| KOR_09 | TT | Non-response | - | 325 |
| KOR_10 | TT | Non-response | - | 517 |
| KOR_11 | TT | Non-response | - | 422 |
| KOR_12 | TT | Non-response | - | 924 |
| KOR_13 | TT | Non-response | - | 902 |
| KOR_14 | TT | Non-response | - | 420 |
| KOR_15 | TT | Non-response | - | 544 |
| CEU_01 | GG | Response | 451 | 3769 |
| CEU_02 | GG | Response | 406 | 3255 |
| CEU_03 | GG | Response | 456 | 1240 |
| CEU_04 | GG | Response | 361 | 3433 |
| CEU_05 | GG | Response | 279 | 3646 |
| CEU_06 | GG | Response | 440 | 3674 |
| CEU_07 | GG | Response | 462 | 2100 |
| CEU_08 | GG | Response | 310 | 1682 |
| CEU_09 | TT | Non-response | - | 2343 |
| CEU_10 | TT | Non-response | - | 1432 |
| CEU_11 | TT | Non-response | - | 3567 |
| CEU_12 | TT | Non-response | - | 3772 |
| CEU_13 | TT | Non-response | - | 2402 |
| CEU_14 | TT | Non-response | - | 2057 |
| CEU_15 | TT | Non-response | - | 3292 |

DMR, Deep molecular response.

^§^TT indicates TCGAATAC/TCGAATAC homozygote haplotype. GG indicates GTCTGCGT/GTCTGCGT homozygote haplotype.

^†^Achievement of DMR indicates whether or not achievement of DMR following imatinib therapy.

^‡^Achieving duration of DMR was defined as the interval from first treatment to date of achieving DMR or the last follow-up date if not met the criteria for response of DMR.

^¶^Follow-up duration was defined as the interval between first treatment and the date of last follow-up.

**Table S7. The types of *HMGCLL1* alternative isoform based on public databases (NCBI and Ensembl)**

| **Alternative isoforms** | **RefSeq ID** | **Ensembl Transcript ID** |
| --- | --- | --- |
| *HMGCLL1*-IS1 | NM_019036 | ENST00000398661 |
| *HMGCLL1*-IS2 | NM_001042406 | ENST00000274901 |
| *HMGCLL1*-IS3 | NM_001287741 | ENST00000308161 |
| *HMGCLL1*-IS4 | NM_001287753 | ENST00000370850 |
| *HMGCLL1*-IS5 | NR_109869 | ENST00000508459 |
| *HMGCLL1*-IS6 | NR_109867 | ENST00000428842 |

**Table S8. The expression level of splicing transcript type of *HMGCLL1* is associated with TCGAATAC haplotype identified by expression quantitative trait loci analysis.**

The expression level is presented the minus ΔCt (*GAPDH* Ct value - *HMGCLL1* Ct value) value for six types (*HMGCLL1* total gene, IS1+IS2, IS3+IS6, IS4, IS5, and IS6). The data was displayed as box-and-whisker plot in Supplementary Fig. 5.

| **ID** | **Genotype^§^** | **Total Gene** | ***HMGCLL1*-IS1+IS2** | ***HMGCLL1*-IS3+IS6** | ***HMGCLL1*-IS4** | ***HMGCLL1*-IS5** | ***HMGCLL1*-IS6** |
| --- | --- | --- | --- | --- | --- | --- | --- |
| 1 | GG | -15.12 | -12.84 | -16.23 | -15.81 | -16.01 | -14.72 |
| 2 | TT | -14.34 | -13.74 | -15.19 | -14.05 | -12.59 | -15.44 |
| 3 | GG | -14.08 | -14.97 | -15.11 | -13.82 | -12.61 | -14.68 |
| 5 | TT | -13.40 | -13.08 | -15.53 | -14.39 | -10.74 | -14.78 |
| 6 | GG | -14.53 | -13.55 | -15.72 | -13.80 | -9.36 | -15.82 |
| 7 | TG | -13.51 | -12.40 | -16.31 | -14.25 | -18.02 | -15.11 |
| 8 | TG | -14.51 | -14.17 | -15.65 | -16.90 | -15.72 | -16.21 |
| 9 | TT | -13.37 | -11.25 | -14.91 | -13.73 | -12.26 | -14.82 |
| 10 | TG | -14.58 | -14.90 | -15.45 | -13.64 | -16.17 | -16.93 |
| 11 | TG | -15.18 | -15.30 | -16.89 | -13.90 | -14.11 | -16.86 |
| 12 | TT | -14.25 | -13.95 | -14.34 | -13.08 | -15.64 | -16.04 |
| 13 | TG | -14.51 | -15.84 | -15.02 | -16.59 | -15.21 | -17.82 |
| 14 | GG | -14.91 | -12.73 | -16.06 | -15.23 | -12.75 | -14.70 |
| 15 | TG | -14.10 | -12.40 | -15.12 | -14.86 | -12.69 | -14.12 |
| 16 | TG | -15.37 | -13.91 | -16.10 | -15.51 | -15.11 | -15.88 |
| 17 | TG | -13.55 | -14.92 | -14.52 | -14.34 | -12.03 | -13.96 |
| 18 | TG | -15.37 | -13.71 | -15.42 | -14.46 | -13.66 | -14.97 |
| 19 | GG | -15.01 | -13.23 | -16.50 | -15.61 | -12.59 | -15.39 |
| 20 | GG | -13.97 | -12.47 | -14.56 | -12.05 | -12.41 | -13.84 |
| 21 | GG | -14.01 | -11.15 | -13.31 | -13.08 | -11.87 | -12.53 |
| 22 | GG | -16.27 | -13.78 | -16.79 | -16.48 | -18.15 | -16.45 |
| 23 | GG | -13.89 | -12.51 | -15.35 | -15.24 | -15.49 | -14.21 |
| 24 | TG | -14.98 | -14.94 | -15.72 | -13.55 | -16.15 | -16.26 |
| 25 | TG | -13.49 | -12.00 | -13.73 | -13.48 | -11.71 | -13.30 |
| 26 | TG | -14.12 | -12.94 | -16.11 | -14.25 | -12.86 | -14.78 |
| 27 | TG | -14.57 | -13.24 | -15.03 | -15.84 | -13.59 | -14.93 |
| 28 | TG | -15.17 | -13.31 | -14.67 | -13.20 | -13.15 | -15.55 |
| 29 | TG | -14.47 | -13.54 | -16.39 | -14.87 | -13.57 | -15.03 |
| 30 | TG | -15.32 | -12.65 | -15.82 | -14.65 | -14.52 | -14.95 |
| 31 | GG | -15.51 | -14.17 | -17.78 | -16.06 | -16.28 | -13.61 |
| 32 | TG | -15.03 | -14.09 | -15.66 | -14.91 | -15.17 | -15.58 |
| 33 | TG | -13.75 | -13.02 | -15.62 | -13.74 | -14.98 | -14.37 |
| 34 | TG | -15.45 | -13.28 | -16.28 | -15.63 | -14.11 | -14.50 |
| 35 | GG | -14.81 | -13.12 | -16.17 | -14.00 | -12.99 | -14.28 |
| 36 | TG | -15.31 | -13.77 | -15.73 | -14.40 | -15.66 | -14.00 |
| 37 | GG | -16.20 | -17.11 | -16.92 | -15.40 | -18.13 | -15.71 |
| 38 | TG | -15.26 | -12.86 | -16.40 | -15.61 | -12.12 | -14.68 |
| 39 | TG | -13.34 | -13.64 | -14.55 | -13.64 | -10.97 | -13.20 |
| 40 | TG | -14.90 | -13.83 | -15.98 | -14.62 | -16.95 | -15.71 |
| 41 | TT | -14.14 | -13.19 | -15.89 | -14.47 | -14.34 | -15.18 |
| 42 | TG | -14.74 | -14.20 | -16.06 | -14.51 | -13.91 | -14.67 |
| 43 | GG | -14.16 | -13.22 | -15.34 | -13.78 | -13.67 | -14.43 |
| 44 | GG | -14.60 | -13.03 | -15.96 | -15.35 | -13.46 | -14.83 |
| 46 | TG | -12.63 | -14.87 | -14.42 | -13.29 | -14.49 | -12.27 |
| 47 | TT | -12.03 | -12.85 | -12.50 | -10.93 | -13.04 | -10.25 |
| 48 | TG | -15.08 | -13.41 | -16.79 | -15.21 | -14.26 | -15.54 |
| 49 | TG | -14.04 | -12.78 | -14.64 | -14.38 | -13.90 | -15.21 |
| 50 | TG | -13.96 | -12.93 | -15.67 | -13.70 | -12.56 | -15.84 |
| 51 | TG | -13.76 | -12.77 | -15.36 | -14.07 | -13.38 | -15.03 |
| 52 | TG | -13.17 | -12.59 | -14.52 | -13.15 | -13.03 | -14.85 |
| 53 | TG | -13.18 | -12.75 | -15.25 | -13.24 | -13.49 | -15.26 |
| 54 | TG | -12.55 | -11.61 | -13.95 | -12.86 | -11.97 | -14.32 |
| 55 | GG | -13.36 | -12.45 | -14.93 | -13.29 | -13.28 | -14.75 |
| 56 | GG | -15.56 | -11.96 | -15.36 | -13.35 | -13.70 | -14.59 |
| 57 | TG | -12.30 | -12.67 | -14.63 | -12.70 | -12.29 | -14.64 |
| 58 | TT | -11.79 | -11.95 | -14.17 | -11.59 | -12.59 | -13.75 |
| 59 | TG | -13.25 | -12.99 | -14.82 | -13.92 | -13.21 | -15.64 |
| 60 | GG | -15.14 | -13.69 | -16.33 | -14.22 | -12.39 | -16.40 |
| 61 | GG | -14.10 | -15.20 | -14.96 | -12.29 | -18.04 | -15.99 |
| 62 | TG | -12.74 | -14.19 | -12.81 | -11.36 | -15.50 | -14.55 |
| 63 | TT | -13.65 | -14.54 | -14.72 | -13.15 | -15.83 | -16.50 |
| 64 | TG | -15.21 | -16.85 | -15.19 | -13.09 | -16.12 | -16.49 |
| 65 | GG | -14.60 | -15.14 | -15.63 | -14.77 | -18.71 | -15.80 |
| 66 | TG | -14.89 | -13.92 | -15.13 | -13.64 | -18.93 | -15.93 |
| 67 | TG | -14.88 | -15.04 | -14.87 | -12.61 | -17.72 | -14.67 |
| 68 | TT | -14.62 | -14.49 | -15.37 | -14.39 | -16.26 | -15.72 |
| 69 | GG | -13.76 | -15.03 | -14.39 | -12.28 | -16.69 | -15.69 |
| 70 | TG | -14.07 | -15.37 | -15.81 | -14.13 | -18.66 | -14.57 |
| 71 | TT | -14.33 | -15.20 | -15.61 | -13.38 | -15.74 | -15.83 |
| 72 | GG | -14.13 | -14.18 | -14.44 | -12.80 | -17.71 | -15.29 |
| 73 | GG | -16.16 | -14.29 | -16.58 | -13.65 | -19.79 | -15.48 |
| 74 | TG | -15.41 | -14.71 | -14.75 | -13.10 | -18.79 | -16.50 |
| 75 | TG | -13.91 | -15.15 | -14.22 | -12.84 | -17.55 | -14.30 |
| 76 | TG | -13.33 | -14.76 | -14.27 | -11.87 | -16.51 | -13.77 |
| 77 | GG | -11.35 | -13.89 | -15.16 | -12.99 | -17.05 | -15.69 |
| 78 | TT | -13.12 | -14.52 | -14.66 | -14.25 | -16.15 | -15.46 |
| 79 | GG | -15.27 | -14.80 | -14.64 | -14.38 | -17.27 | -14.99 |
| 80 | TG | -14.61 | -14.76 | -15.22 | -13.22 | -19.43 | -15.36 |
| 81 | TG | -15.08 | -15.54 | -14.60 | -14.12 | -19.59 | -15.80 |
| 82 | GG | -16.45 | -15.85 | -15.87 | -14.26 | -19.05 | -15.93 |
| 83 | TG | -14.93 | -16.50 | -15.31 | -13.69 | -18.01 | -14.67 |
| 84 | GG | -15.33 | -15.19 | -14.90 | -12.23 | -15.83 | -15.72 |
| 85 | GG | -14.82 | -13.83 | -14.53 | -14.80 | -14.21 | -15.69 |
| 86 | TG | -15.88 | -15.61 | -14.59 | -13.37 | -17.71 | -14.57 |
| 87 | GG | -16.06 | -16.27 | -15.61 | -13.89 | -18.56 | -15.83 |
| 88 | GG | -15.25 | -15.21 | -13.86 | -12.17 | -15.67 | -15.29 |
| 89 | GG | -14.55 | -15.83 | -15.28 | -14.78 | -16.56 | -15.48 |
| 90 | GG | -16.69 | -15.65 | -15.74 | -13.28 | -19.48 | -16.50 |
| 91 | GG | -16.69 | -15.10 | -16.00 | -13.88 | -14.64 | -14.30 |
| 92 | TG | -15.04 | -14.45 | -14.01 | -11.17 | -16.96 | -13.77 |
| 93 | TT | -15.38 | -15.12 | -15.52 | -13.74 | -15.48 | -15.69 |
| 94 | TG | -15.01 | -16.57 | -15.78 | -13.40 | -19.69 | -15.46 |
| 95 | GG | -15.78 | -14.98 | -13.76 | -12.42 | -14.89 | -14.99 |
| 96 | TT | -15.14 | -14.08 | -13.64 | -11.08 | -15.86 | -15.36 |
| 97 | TG | -15.30 | -16.07 | -15.42 | -15.66 | -17.77 | -16.89 |
| 98 | TT | -15.03 | -14.61 | -15.06 | -13.26 | -15.11 | -15.98 |
| 99 | GG | -14.74 | -16.56 | -16.07 | -13.55 | -15.81 | -16.42 |
| 100 | TT | -15.21 | -14.09 | -15.34 | -13.72 | -14.61 | -12.80 |
| 101 | TT | -14.94 | -15.03 | -14.58 | -13.55 | -15.12 | -16.11 |
| 102 | GG | -15.47 | -14.59 | -14.67 | -12.35 | -14.19 | -16.37 |
| 103 | TG | -15.16 | -15.97 | -14.53 | -13.03 | -15.96 | -16.54 |
| 104 | TG | -14.77 | -17.27 | -15.34 | -15.65 | -19.80 | -15.28 |
| 105 | GG | -14.48 | -14.19 | -13.21 | -13.71 | -13.90 | -14.42 |
| 106 | TT | -14.38 | -14.37 | -14.88 | -13.13 | -15.55 | -15.45 |
| 107 | TT | -13.97 | -14.65 | -13.75 | -12.43 | -14.70 | -14.34 |
| 108 | TG | -13.41 | -14.28 | -16.00 | -14.01 | -15.19 | -14.01 |
| 109 | GG | -13.76 | -16.32 | -15.05 | -13.94 | -18.49 | -15.60 |
| 110 | TG | -15.47 | -16.43 | -15.65 | -15.48 | -17.34 | -16.86 |
| 111 | GG | -15.81 | -14.82 | -15.99 | -15.52 | -14.13 | -14.33 |
| 112 | TG | -15.43 | -16.11 | -9.81 | -8.14 | -15.58 | -16.87 |

^§^TT indicates the TCGAATAC/TCGAATAC homozygote haplotype. TG represents TCGAATAC/GTCTGCGT heterozygote haplotype. GG indicates the GTCTGCGT/GTCTGCGT homozygote haplotype.

**Table S9. Results of cell viability assay using various siRNAs to interfere specific isoforms in K562 cell line at 72h.**

| **Isoforms** | **siRNA names** | **siRNA target sites** | **Relative cell viability (%)*** |
| --- | --- | --- | --- |
| Control | - | - | 100 ± 2.0 |
| Negative control | Non-silencing siRNA | - | 97.5 ± 8.9 |
| *HMGCLL1*-IS1 | IS1si-1 | Exon 2-Exon 3 junction | 99.3 ± 1.4 |
|  | IS1si-2 | Exon 2 | 99.7 ± 1.2 |
| *HMGCLL1*-IS1+2 | IS12si-1 | Exon 5 | 92.8 ± 1.8 |
|  | IS12si-2 | Exon 5 | 96.0 ± 5.4 |
| *HMGCLL1*-IS3 | IS3si | Exon 6-Exon 7 Junction | 32.6 ± 10.0 |
| *HMGCLL1*-IS4 | IS4si-1 | Exon 4-Exon 8 Junction | 92.1 ± 2.1 |
|  | IS4si-2 | Exon 4-Exon 8 Junction | 96.3 ± 2.9 |
| *HMGCLL1*-IS5 | IS5si-1 | Exon 4-Exon 9 Junction | 97.5 ± 1.9 |
|  | IS5si-2 | Exon 4-Exon 9 Junction | 98.7 ± 3.6 |
| *HMGCLL1*-IS6 | IS6si-1 | Exon 6-UTR Junction | 99.6 ± 1.5 |
|  | IS6si-2 | Exon 6-UTR Junction | 93.3 ± 5.4 |

siRNA, small interfering RNA; IS, isoform.

^*^Relative Cell viability (%) was calculated as (absorbance of the treated wells - absorbance of the blank wells)/ (absorbance of the control wells - absorbance of the blank wells) at 72h time point.

**Table S10. RNA sequencing results show that *HMGCLL1* blockade using IS3 siRNA can downregulate cell cycle mediated genes**

The table shows a list of the top 11 genes with PPDE = 1 and |log2 FC| > 2 marked with a red dot in the Fig. 6a. Seven of these 11 genes were downregulated while 4 genes were upregulated after IS3 blockade.

| **Gene (Ensembl ID_GeneSymbol)** | **PPEE** | **PPDE** | **Log2 Fold Change** |
| --- | --- | --- | --- |
| ENSG00000258017_*RP11-386G11.10* | 0 | 1 | -3.62 |
| ENSG00000181061_*HIGD1A* | 0 | 1 | -2.56 |
| ENSG00000135446_*CDK4* | 0 | 1 | -2.39 |
| ENSG00000213465_*ARL2* | 0 | 1 | -2.28 |
| ENSG00000105810_*CDK6* | 0 | 1 | -2.13 |
| ENSG00000238227_*C9orf69* | 0 | 1 | -2.09 |
| ENSG00000141101_*NOB1* | 0 | 1 | -2.07 |
| ENSG00000114270_*COL7A1* | 0 | 1 | 2.12 |
| ENSG00000140678_*ITGAX* | 0 | 1 | 2.17 |
| ENSG00000259207_*ITGB3* | 0 | 1 | 2.44 |
| ENSG00000196878_*LAMB3* | 0 | 1 | 2.55 |

PPEE, posterior probability differentially expressed; PPDE, posterior probability equally expressed.

**Table S11. Detailed enrichment results by gene set enrichment analysis (GSEA) compared to control and IS3 blockade**

GSEA was performed with 12 134 genes marked by black and red dots in the Fig. 6a. Gene sets were defined by the KEGG pathway database downloaded from the Broad ftp website. **a** Two gene sets were significantly upregulated in the control group with FDR q-value < 0.05. **b** Eight gene sets were significantly upregulated in IS3si treated group with FDR q-value < 0.05.

**a**

| Rank | Name | NES | NOM p-val | FDR q-val | FWER p-val |
| --- | --- | --- | --- | --- | --- |
| 1 | KEGG_CELL_CYCLE | 2.14 | < .001 | .001 | .001 |
| 2 | KEGG_SPLICEOSOME | 1.78 | < .001 | .044 | .103 |

**b**

| Rank | Name | NES | NOM p-val | FDR q-val | FWER p-val |
| --- | --- | --- | --- | --- | --- |
| 1 | KEGG_ECM_RECEPTOR_INTERACTION | -2.38 | < .001 | < .001 | < .001 |
| 2 | KEGG_HEMATOPOIETIC_CELL_LINEAGE | -2.36 | < .001 | < .001 | < .001 |
| 3 | KEGG_HYPERTROPHIC_CARDIOMYOPATHY_HCM | -1.98 | < .001 | .015 | .020 |
| 4 | KEGG_DILATED_CARDIOMYOPATHY | -1.91 | < .001 | .017 | .030 |
| 5 | KEGG_CYTOKINE_CYTOKINE_RECEPTOR_INTERACTION | -1.86 | < .001 | .021 | .045 |
| 6 | KEGG_CELL_ADHESION_MOLECULES_CAMS | -1.84 | < .001 | .019 | .048 |
| 7 | KEGG_METABOLISM_OF_XENOBIOTICS_BY_CYTOCHROME_P450 | -1.83 | .005 | .018 | .053 |
| 8 | KEGG_FOCAL_ADHESION | -1.76 | < .001 | .025 | .084 |

NES=Normalized Enrichment Score. NOM p-val=Nominal *p* value. FDR q-val=False discovery rate q value. FWER p-val=Familywise-error rate *p* value.

**Table S12. Primer and siRNA sequences used for *in vitro* assay**

| **Type** | **Targeted splicing variant** | **Primer/siRNA name** | **Sense strand** | **Antisense strand** |
| --- | --- | --- | --- | --- |
| qPCR primer | IS1, IS2, IS3, IS4, IS5, IS6 | *HMGCLL1* Total Gene | ATGTGCCATCCGCGGTGAA | TCCCCGATCCAGAGATGCT |
| qPCR primer | IS1, IS2 | *HMGCLL1* IS1+IS2 | TACCACAGATGGCTGATCACAC | GCATGGTGAAAACCCTGAAGAT |
| qPCR primer | IS3, IS6 | *HMGCLL1* IS3+IS6 | TACCACAGGTTGCTGCTGGAG | TGCAGACTTAACAACCTCCTCAA |
| qPCR primer | IS4 | *HMGCLL1* IS4 | TACCACAGGTGTCTAAGAGATT | GACAGTGAACAGCAAGAGCA |
| qPCR primer | IS5 | *HMGCLL1* IS5 | TACCACAGATGGGAATTAATGT | AATCCTCAGTGGCTACATTCC |
| qPCR primer | IS6 | *HMGCLL1* IS6 | TACCACAGGTTGCTGCTGGAG | TCATAAGTACCCTCGTGCTGGA |
| siRNA | IS3, IS6 | IS3si-1 | GGUUGCUGCUGGAGCUACUGA (dTdT) | UCAGUAGCUCCAGCAGCAACC (dTdT) |
| siRNA | IS3, IS6 | IS3si-2 | GGUACCACAGGUUGCUGCU(dTdT) | AGCAGCAACCUGUGGUACC(dTdT) |

**Figure S1. Regional plot of candidate regions in the discovery set.**

**a** and **b** show regional association plots of selected as candidate loci in which more than 5 SNPs with *p* < 10^-4^ within 1Mb are observed and minimum *p* <5.0 x 10^-5^ in discovery set. 6p12.1 locus was validated in independent validation set. The purple shaded circle represents rs9370435 (located in the intron of *HMGCLL1* in the plot (A) or rs424280 (the most significant SNP in 16q23.3 in the plot **b**. Candidate genes included *HMGCLL1, GFRAL,* and *BMP5* near the 6p12.1 locus and *CDH13* and *HSBP1* near the 16q23.3 locus. ○ represents genotyped SNPs and □ indicates imputed SNPs in the discovery set. The blue line indicates the recombination rate while filled color represents the linkage disequilibrium score based on r^2^ values estimated from the 1000 genome Mar 2012 ASN data.

**A** 6p12.1

**B** 16q23.3

**Figure S2. LD block patterns within the validated gene, *HMGCLL1*, in East Asian and European population.**

Visualization of LD (r^2^) was generated in Haploview using HapMap project genotype data (Phase II Public Release #22 NCBI Build 36). Darker color indicates a higher LD score (r^2^) among SNPs. Black color indicates r^2^ = 1. **a** LD block pattern in the CHB+JPT (East Asian) population. **b** LD block pattern in the CEU (European) population. The red line indicates the exonic region of *HMGCLL1*. Exon 6 and exon 7 are marked by red arrows. With respect to its direction, *HMGCLL1* is a reverse strand gene. We used the Asian population in the discovery set and the European population in the validation set.

**A** CHB+JPT (East Asian) population


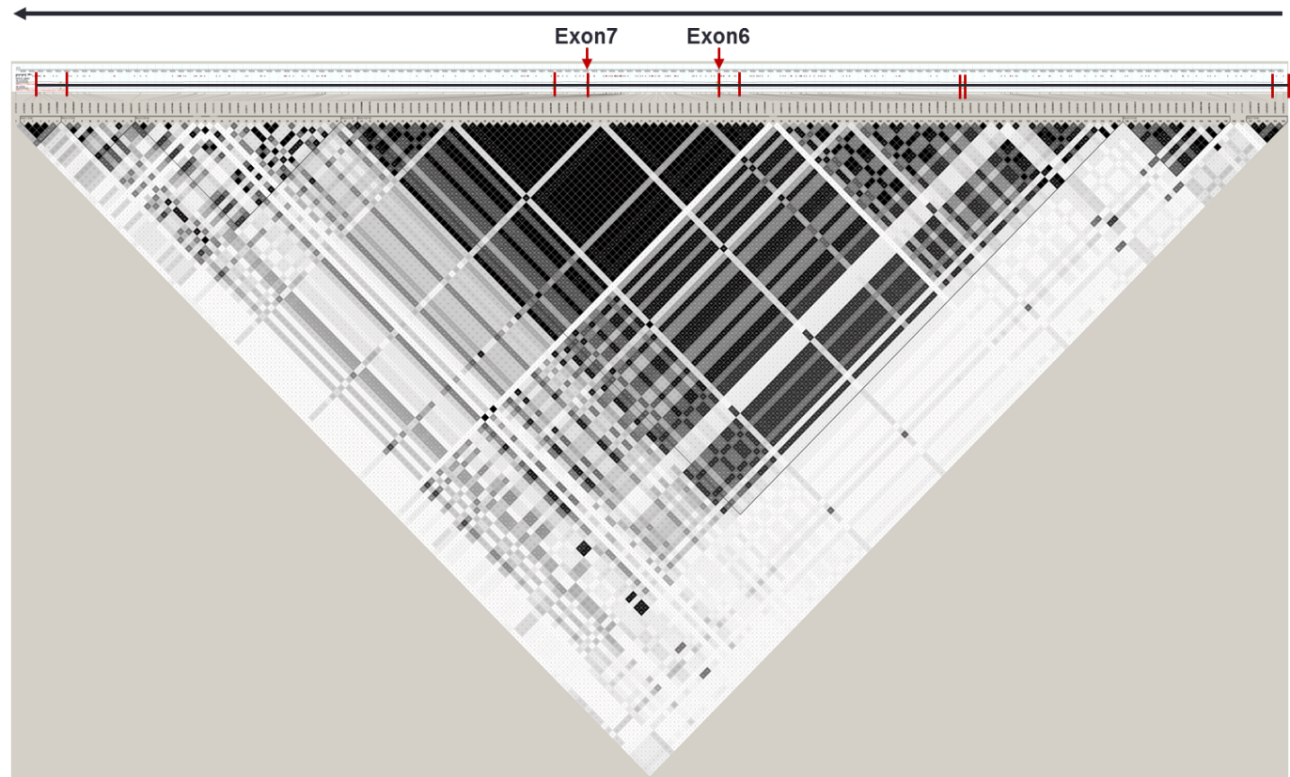


**B** CEU (European) population


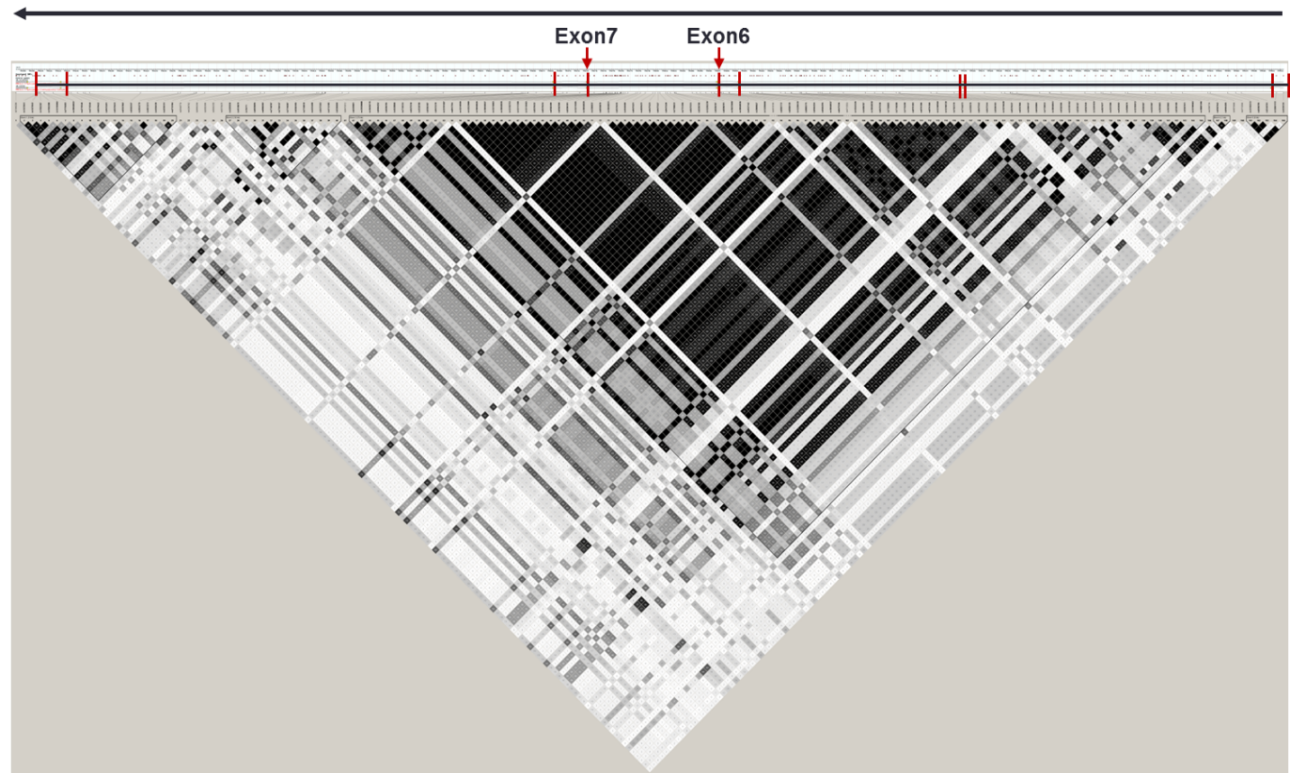


**Figure S3. Cumulative incidence plot of deep molecular response (DMR) in only chronic phase (CP) patients in the discovery and validation sets, respectively.**

**a** and **b** The plots show cumulative incidence of deep molecular response (DMR) in only chronic phase (CP) patients in the discovery set (n=182) and in the validation set (n=241), respectively. The red line indicates the group with TCGAATAC. TT indicates TCGAATAC/TCGAATAC homozygote haplotype. TG represents TCGAATAC/GTCTGCGT heterozygote haplotype. GG indicates GTCTGCGT/GTCTGCGT homozygote haplotype. One case in the discovery set and three cases in the validation set did not have haplotype information due to missing data of genotype or different haplotype constructed.

**Figure S4. Cumulative incidence plot of major molecular response (MMR) in the discovery and validation sets, respectively.**

**a** and **b** The plots show cumulative incidence of major molecular response (MMR) in the discovery set and in the validation set, respectively. The red line indicates the group with TCGAATAC. TT indicates TCGAATAC/TCGAATAC homozygote haplotype. TG represents TCGAATAC/GTCTGCGT heterozygote haplotype. GG indicates GTCTGCGT/GTCTGCGT homozygote haplotype.

**Figure S5. Splicing transcript types of *HMGCLL1* and eQTL results associated with TCGAATAC haplotype.**

**a** The plot shows isoform structures of *HMGCLL1* referred from public databases (NCBI and Ensembl). The location of validated TCGAATAC haplotype is within intron 6 (between exon 6 and exon 7) of *HMGCLL1*. Red arrows indicate forward primer binding sites. Green arrows indicate reverse primer binding sites. **b** The plot shows the eQTL signal of homogeneous haplotypes associated with DMR following IM therapy. Minus ΔCt (*GAPDH* Ct value - *HMGCLL1* Ct value) indicates expression level (Y axis) by haplotype (X axis) for six types (*HMGCLL1* total gene, IS1+IS2, IS3+IS6, IS4, IS5, and IS6) using box-and-whisker plot. IS1+IS2 indicates that IS1 and IS2 are simultaneously measured due to sequence homology of the target isoform. IS3 + IS6 indicates that IS3 and IS6 are simultaneously measured due to sequence homology. Because IS6 was not detected by eQTL using the IS6 specific qPCR primer, the eQTL result of IS3+IS6 reflected gene expression of IS3 only, which showed significant association with the haplotype. **c** The plot shows the eQTL signal of each haplotype associated with DMR following IM therapy. Statistical analysis was performed by Wilcoxon rank sum test **b** and Kruskal-Wallis rank sum test **c**. TT indicates the TCGAATAC/TCGAATAC homozygote haplotype. TG represents TCGAATAC/GTCTGCGT heterozygote haplotype. GG indicates the GTCTGCGT/GTCTGCGT homozygote haplotype. ** *p* < 0.01, * *p* < 0.05.

**A**

**B**

**C**

**Figure S6. Splicing transcript types of *HMGCLL1* and targeted siRNA binding sites to interfere each isoform.**

**a** Some siRNAs were designed for exon-exon junction to target each isoform specific site. Red lines indicate siRNA binding sites to target each isoform. **b** The plot shows that relative expression of total *HMGCLL1* and IS3 measured using qPCR in K562 cell line transfected with IS3 siRNA at 24h time point. Decreased expression level of total *HMGCLL1* and IS3 after IS3si treatment was confirmed using qPCR in K562 cell line. Relative mRNA expression was calculated with the delta-delta Ct equation using *GAPDH* as a reference gene. All data in the bar graph are presented as average ± standard deviation from at least three independent wells. The results show are representative of at least three independent experiments. Statistical analysis was performed using Student’s t-test with equal variance. **c** Sequence homology of *HMGCLL1*-IS3 cDNAs between human and murine genomes. Human and murine cDNA sequences were downloaded from public databases (Ensembl and NCBI). The red line indicates the IS3si binding site. IS3si designed for human could be applied to BaF3 cells derived from murine using the same protocol for human cell lines. The sequence marked by asterisk is a perfect match between human and murine.

**A**

**
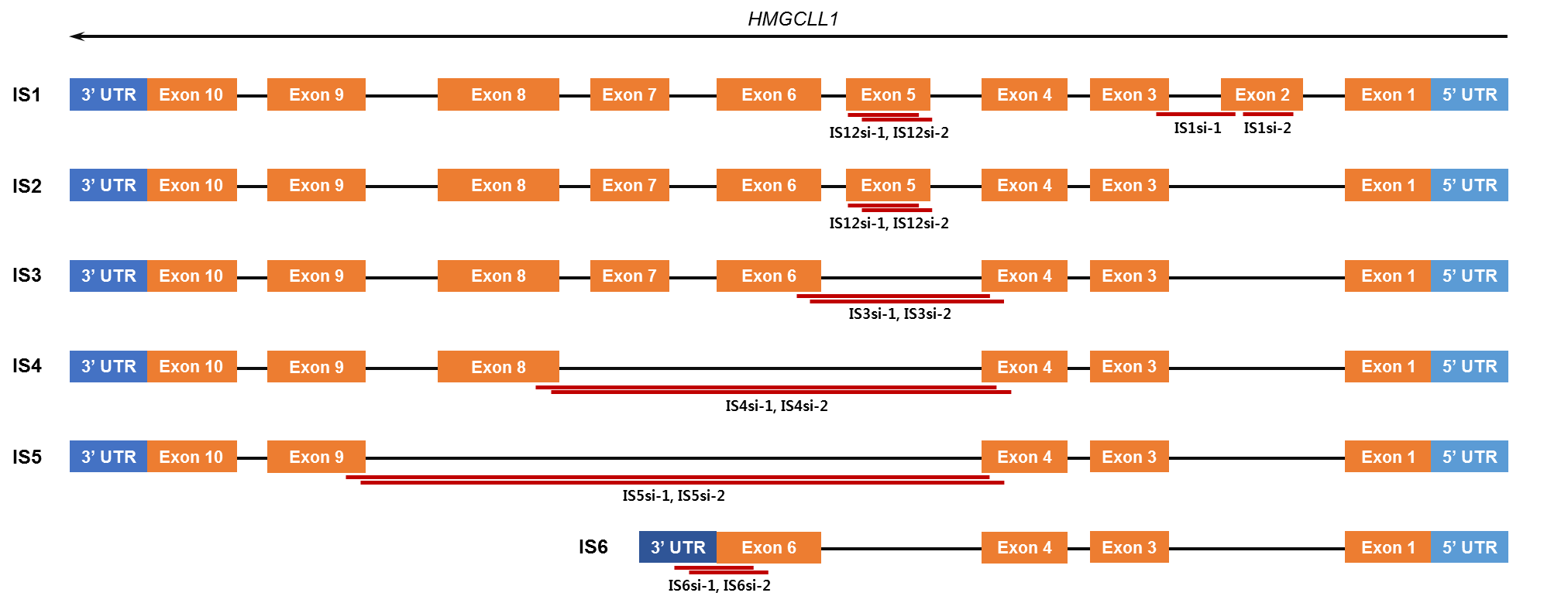
**

**B C**


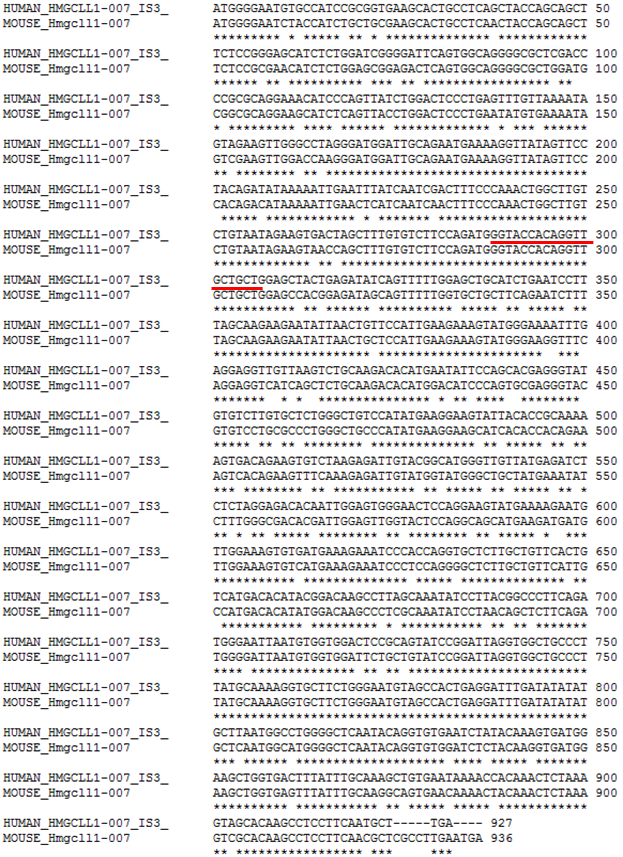


**Figure S7. Phospho-CrkL (pCrkL)/CrkL ratio assessed as *BCR-ABL1* activity in K562, BaF3/WT, and BaF3/T315I cell lines.**

**a-c** To measure the phospho-CrkL/CrkL ratio, cells transfected with IS3si with or without treatment with TKIs were assayed using the Phospho-CrkL (Tyr207) colorimetric cell-based enzyme linked immunosorbent assay kit. The Phospho-CrkL/CrkL ratio relative to control (Y axis) is presented as average ± standard deviation from at least three independent wells measured by colorimetric cell-based assay at the 48h time point in K562, BaF3/WT, and BaF3/T315I*^mut^* cells. Statistical analysis was performed using Student’s *t*-test with equal variance. *** *p* < 0.001.

**Figure S8. Confirmation of acquired heterozygous T315I mutation in K562 cell line using genome editing technology based on CRISPR/Cas9 system.**

We performed genome editing with the CRISPR/Cas9 system, using the Cas9 RNA guided DNA endonuclease. The upper panel indicates the normal K562 cell line sequence. The lower panel indicates the target mutation (c.944C>T) sequence generated in K562/T315I*^mut^* cells which was confirmed by capillary sequencing.


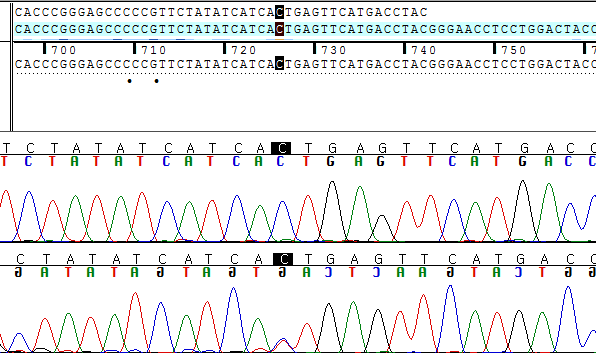


**Figure S9. Decreased expression level of *CDK4* and *CDK6* after IS3si treatment was confirmed using qPCR in K562 and LAMA84 cell lines.**

**a** and **b** The plots show that relative expression of *CDK4* and *CDK6* was measured using qPCR in **a** K562 and **b** LAMA84 *BCR-ABL1*-positive cell lines transfected with IS3 siRNA at 24h time point. Relative mRNA expression was calculated with the delta-delta Ct equation using *GAPDH* as a reference gene. All data in the bar graph are presented as average ± standard deviation from at least three independent wells. The results show are representative of at least three independent experiments. Statistical analysis was performed using Student’s t-test with equal variance at the 72h. *** *p* < 0.001.
